# Correction: Isolation and purification of DNA double-strand break repair intermediates for understanding complex molecular mechanisms

**DOI:** 10.1371/journal.pone.0331774

**Published:** 2025-09-05

**Authors:** The PLOS ONE Staff

## Notice of republication

This article was republished on August 21, 2025, to correct an error in Figures 3, 6, and 9, and an error in the body text. Incorrect versions of Figures 3, 6, and 9 were published in error. Additionally, in Section 3.2, there was an error in the second sentence of the first paragraph.

The publisher apologizes for the error. Please download this article again to view the correct version. The originally published, uncorrected article and the republished, corrected article are provided here for reference.

## Supporting information

S1 File Originally published uncorrected article. (PDF)

S2 File Republished corrected article. (PDF)
