# Supplementary material for: Isolation and purification of DNA double-strand break repair intermediates for understanding complex molecular mechanisms
Source: PLoS One. 2024 Oct 11;19(10):e0308786. doi: 10.1371/journal.pone.0308786 (PMC11469543; doi:10.1371/journal.pone.0308786)
Supplement: S1 File — (DOCX) [file pone.0308786.s001.docx]

**Supplementary information S1: Construction and integration of a 40 kb synthetic region of DNA**

A 40 kb region of chromosomal DNA was designed to be free of several 4-base restriction enzyme target sites, a property that could be used to fragment the rest of the genome. The list of the REs is given below:

AfaI (Csp6I, CviQI, RsaI, RsaNI), MnlI, AccII (Bsh1236I, BspFNI, BstFNI, BstUI, MvnI), AspLEI (BstHHI, CfoI, GlaI, HhaI, Hin6I, HinP1I, HspAI), HpyCH4V, HpyCH4IV (HpySE526I, MaeII, TaiI) and BfaI (FspBI, MaeI, XspI).

In addition, a kanamycin resistance gene was included to enable selection for chromosomal integration and restriction target sites were placed at specific locations to facilitate further analysis. The list of the REs whose recognition sites have been inserted in the synthetic DNA is given below:

SalI, PstI, NotI, BamHI, BglII, AvaII, SacI, SmaI, NcoI, and ISceI.

The designed sequence was synthesized and integrated into the *E. coli* genome before the insertion of the 246 bp interrupted palindrome required to generate the DSB as this facilitated its construction and chromosomal insertion and promoted future flexibility regarding the nature and precise location of the DSB site. Triple Chi arrays (recognized by RecBCD to initiate recombination) were designed 4 kb away on either side of the intended DSB site. In addition to the 40 kb region and the kanamycin gene, 5 kb homology arms, identical to the *E. coli* genome on either side of the 40 kb region, were included in the synthetic DNA design to facilitate its integration into the *E. coli* chromosome; the end design construct being a 51 kb DNA sequence. The 51 kb sequence was flanked by PstI restriction sites to allow it to be released from the final vector when required.

The 51 kb synthetic construct, consisting of the designed 40 kb region, the kanamycin resistance gene (*Kan^R^*), and the 5kb homology arms, was synthesized in eight approximately equal fragments, with 100 bp overlapping between each other to facilitate assembly by homologous recombination. DNA synthesis was carried out by WuXi Qinglan Biotechnology Inc. And the assembly was done in the laboratory of Prof. Patrick Cai, initially at the University of Edinburgh and completed at the University of Manchester. In this laboratory a pBUCA receiver vector (pSJ034) was adapted to serve as a low copy number backbone for the DNA assembly. A gBlock containing regions of homology to the start of fragment 1 and end of fragment 8 separated by an SfoI restriction site was cloned into the pBUCA vector replacing the *Kan^R^* gene of the pBUCA plasmid by digestion/ligation. SfoI (NEB, R0606S), a blunt end restriction enzyme for which no sites are present in the remainder of the pBUCA plasmid, was used to linearize the backbone between the homologous regions for fragments 1 and 8. Linearised fragments and backbone were assembled via homologous recombination in yeast strain BY4741 [1]. Colonies were initially screened for plasmids with the correct assembly using PCR at the junctions between fragments and/or backbone. The primers are listed in supplementary table S3. Having confirmed the presence of correctly assembled junctions, plasmids were isolated from yeast using a Zymoprep yeast plasmid miniprep II kit (Zymo Research, D2004) according to the manufacturer’s instructions. Promising plasmids were then transformed into *E. coli* for further screening and recovery. Plasmids were first transformed into DH5α and, from DH5α, they were further isolated and transformed into DL7119, a *recA* mutant background, for further screening provided by the Leach laboratory. Finally, sequenced plasmids were provided to the Leach lab as isolated plasmids and as *E. coli* strains in the DL7119 background strain (strain DL7323).

In the Leach lab, initially, the entire 40 kb region to be replaced by the synthetic sequence of DNA was deleted from the *E. coli* chromosome by plasmid mediated gene replacement (PMGR) using plasmid pDL6967, leaving only regions identical to the 5 kb homology arms (DL7008 and DL7011 for the MG1655 and BW27784 background respectively). Integration of the synthetic DNA into the chromosome was done by transforming a *recD* mutant of this Δ40 kb strain (DL7018) with a Pst-I digested linearised sequence containing the synthetic DNA while selecting for kanamycin resistance (DL7394). The synthetic region was then transferred by P1 transduction into a Δ40 kb mutant BW27784 derivative (strain DL7419) to enable controlled expression of SbcCD from the *araBAD* promoter. The use of the Δ40 kb strain removed the risk of recombination within the original 40 kb region during the integration and transfer of the synthetic DNA. The palindrome was inserted using PMGR (strain DL7662) with plasmid pDL7659 containing the 246 bp interrupted palindrome as used in previous studies [2]. pDL7659 was constructed by modifying pDL7431 which contained *lacI* and *lacZ* synthetic alleles as homology arms for chromosome integration. The *ruvAB* mutant was generated by PMGR between this strain (DL7662) and pDL2757.

Construction of plasmid pDL6967: Plasmid pDL6967 was used to introduce a 41 Kb deletion on the *E. coli* chromosome around the *lacZ* gene. Two amplified fragments were digested with SalI and PstI and ligated, after PCR-mediated coupling, into pTOF24. The upstream fragment (404 bp) was amplified from the MG1655 strain using primers DLS1del.F1 and DLS1del.R1. The downstream fragment (449 bp) was amplified from the same strain using primers DLS1del.F2 and DLS1del.R2. The *E. coli* chromosome deletion goes from gene *yahN* to *tauA* (position 344,890-385,418).

Construction of plasmid pDL7431: Plasmid pDL7431 was created to insert a palindrome in the synthetic DNA generated in this work. Its main feature was to have *lacI* and *lacZ* synthetic alleles as homology arms for chromosome integration. Two amplified fragments were digested with SalI and PstI and ligated, after PCR-mediated coupling, into pTOF24. The upstream fragment (433 bp) was amplified from pSJ034 using primers SynLacZ.AFw and SynLacZ.ARv. The downstream fragment was amplified from pSJ034 with primers SynLacZ.BFw and SynLacZ.BRv. The resulting insert includes the end sequence of lacI and the beginning sequence of lacZ without the 4bp base cutters and other properties described on the synthetic DNA construction.

Construction of plasmid pDL7659: Plasmid pDL7659 was used to integrate a 246 bp palindrome into the lacZ on the synthetic DNA region. Using primers Mypal.CR1 and Mypal.CF2, a ~260 bp fragment was amplified containing the 246 bp palindrome from the lambda bacteriophage [3]. The PCR fragment was digested with EcoRI and ligated into the MfeI restriction site of pDL7431. The presence of the palindrome can be verified by PCR using primers Ex-test_F and Ex-test_R.

**References**

1. Brachmann, C.B., Davies, A., Cost, G.J., Caputo, E., Li, J., Hieter, P., and Boeke, J.D., Designer deletion strains derived from Saccharomyces cerevisiae S288C: a useful set of strains and plasmids for PCR-mediated gene disruption and other applications. Yeast, 1998. **14**(2): p. 115-32.

2. Eykelenboom, J.K., Blackwood, J.K., Okely, E., and Leach, D.R., SbcCD causes a double-strand break at a DNA palindrome in the Escherichia coli chromosome. Mol Cell, 2008. **29**(5): p. 644-51.

3. Leach, D.R., Okely, E.A., and Pinder, D.J., Repair by recombination of DNA containing a palindromic sequence. Mol Microbiol, 1997. **26**(3): p. 597-606.
