# Supplementary material for: Isolation and purification of DNA double-strand break repair intermediates for understanding complex molecular mechanisms
Source: PLoS One. 2024 Oct 11;19(10):e0308786. doi: 10.1371/journal.pone.0308786 (PMC11469543; doi:10.1371/journal.pone.0308786)
Supplement: S1 Table — (DOCX) [file pone.0308786.s002.docx]

**S2 Tables: Bacterial Strains, Plasmids and Oligonucleotides used in this study**

**Bacterial strains**

| Strain | Genotype | Source |
| --- | --- | --- |
| BW27784 | Δ(araD-araB)567 Δ(araH-araF)570(::FRT) ΔaraEp-532::FRT φPcp18araE533 Δ(rhaD-rhaB)568 hsdR514ΔlacZ478(::rrnB-3) | [1] |
| DL654 | hsdR2 Zgq-202::Tn10 recD1014 recA::Cm^R^ | [2] |
| DL1777 | lacIq lacZχ- fnr-267 (ΔynaJ ΔydaA Δfrn Δogt ΔabgT ΔabgB ΔabgA ΔabgR ΔydaL ΔydaM ΔydaN ΔdbpA ΔydaO) | [3] |
| DL5217 | BW27784 *mhpC*Δχ *prpE*Δχ *codB*Δχ *cynX*Δχ *mhpA*::χχχ *lacZY*::χχχ *proA*::I-SceI_cs_ tsx*::*I-SceI_cs_ *cynX*::Gm^R^ ΔP_sbcDC_ P_BAD_-*sbcDC* | PMGR with pDL3192, pDL3181, pDL4690, pDL5087, pDL5172, pDL5174, pDL5173 |
| DL7008 | DL1777 Δ(*lacZ*::40 kb) | PMGR with pDL6967 |
| DL7011 | DL5217 Δ(*lacZ*::40 kb) | PMGR with pDL6967 |
| DL7018 | DL7008 Δ(*recD*) | PMGR with pDL2749 |
| DL7119 | DL7008 *recA*::Cm^R^ | P1 transduction from DL654 |
| DL7323 | DL7119 with pSJ034 | This work |
| DL7394 | DL7018 Δ(345286-385307)::syn*lacZ* | This work |
| DL7419 | Δ( 345286-385307)::*synlacZ* mhpA::χχχ lacZY::χχχ tsx::I-SceI_cs_ proA::I-SceI_cs_ PBAD-sbcDC lacZ^+^ lacIq lacZχ- ,cynX::Δchi, codB::Δchi prpE::Δchi mhpC::Δchi | P1 transduction with DL7394 |
| DL7662 | DL7419 syn*lacZ*::pal246 | PMGR with pDL7659 |
| DL7672 | DL7662 Δ*ruvAB* | PMGR with pDL2757 |
| MG1655 | F- λ - ilvG- rfb-50 rph-1 fnr-267 (ΔynaJ ΔydaA Δfrn Δogt ΔabgT ΔabgB ΔabgA ΔabgR ΔydaL ΔydaM ΔydaN ΔdbpA ΔydaO) | [4] |

**Plasmids**

| Plasmid | Brief description | Source |
| --- | --- | --- |
| pTOF24 | pSC101-derived vector. *repA*(Ts) and *sacB* conferring sucrose sensitivity. Cm^R^ Ts Suc^S^ | [5] |
| pSJ034 | pBUCA Plasmid with the synthetic DNA | This work |
| pDL2749 | pTOF24 with Δ*recD* knock-out fragment, Cm^R^ Ts Suc^S^ | [6] |
| pDL2757 | pTOF24 with Δ*ruvAB* knock-out fragment, Cm^R^ Ts Suc^S^ | [3] |
| pDL3181 | pTOF24 mhpA::χχχ | [7] |
| pDL3192 | pTOF24 lacZY::χχχ | [7] |
| pDL4690 | pTOF24 rph+ | [8] |
| pDL5087 | pTOF24 cynX::Δchi | This work |
| pDL5172 | pTOF24 codB::Δchi | This work |
| pDL5173 | pTOF24 mhpC::Δchi | This work |
| pDL5174 | pTOF24 prpE::Δchi | This work |
| pDL6967 | pTOF24 with Δ *lacZ*::40 Kb region, Cm^R^ Ts Suc^S^ | This work |
| pDL7431 | pTOF24 with *lacZ* from pSJ034, Cm^R^ Ts Suc^S^ | This work |
| pDL7659 | pDL7431 plasmid with a 246 bp palindrome inserted into *lacZ*, Cm^R^ Ts Suc^S^ | This work |

**Oligonucleotides**

| Name | Sequence (5’-3’) | Source | Purpose |
| --- | --- | --- | --- |
| SJp035F | GTGGAAGAGATGAAGGTTACGATTGG | This work | PCR of first fragment of synthetic DNA/pBUCA backbone junction to check for the integration of the synthetic DNA into the backbone |
| SJp055R | ccacgccattttcgtagtcaaaacc | This work | PCR of first fragment of synthetic DNA/pBUCA backbone junction to check for the integration of the synthetic DNA into the backbone |
| SJp056F | ggatagcaatacttatggcaaaactgg | This work | PCR of last fragment of synthetic DNA/pBUCA backbone junction to check for the integration of the synthetic DNA into the backbone |
| SJp057R | cctataaacgctacacaggctcc | This work | PCR of last fragment of synthetic DNA/pBUCA backbone junction to check for the integration of the synthetic DNA into the backbone |
| SJp019F | ctatcgtttacgaaggattgccgac | This work | PCR of the junction of 2^nd^ and 3^rd^ fragment of synthetic DNA to check for appropriate assembly |
| SJp020R | ggatgccccagtcaaaggtgg | This work | PCR of the junction of 2^nd^ and 3^rd^ fragment of synthetic DNA to check for appropriate assembly |
| SynLacZ.AFw | TCTCAGCTGCAGAATAATTCCCGTCTGGCC | This work | Construction of plasmid pDL7431 |
| SynLacZ.ARv | GCTATGACCATGCAATTGCGTCGTTTTACAACTTCGT | This work | Construction of plasmid pDL7431 |
| SynLacZ.BFw | TGTAAAACGACGCAATTGCATGGTCATAGCTGTTTCC | This work | Construction of plasmid pDL7431 |
| SynLacZ.BRv | CGTGTCGACGGTGCGGATATCTCGGTA | This work | Construction of plasmid pDL7431 |
| Mypal.CR1 | AAAGAATTCACAACCTGACCCAGCAAAAG | This work | Construction of plasmid pDL7659 |
| Mypal.CF2 | AAAGAATTCATACCCAGATTGCGAACACC | This work | Construction of plasmid pDL7659 |
| Ex-test_F | TTATGCTTCCGGCTCGTATG | [3] | PCR of *lacZ* to check for the presence of a palindrome |
| Ex-test-R | GGCGATTAAGTTGGGTAACG | [3] | PCR of *lacZ* to check for the presence of a palindrome |
| DLS1del.F1 | AAAAACTGCAGCCCCTTCGTAAATCAGGCGT | This work | Construction of plasmid pDL6967 |
| DLS1del.R1 | GTTCACCGGTCGGCGGCGCTTATCTCTTAT | This work | Construction of plasmid pDL6967 |
| DLS1del.F2 | AGCGCCGCCGACCGGTGAACAAAGCGATCA | This work | Construction of plasmid pDL6967 |
| DLS1del.R2 | AAAAAGTCGACGTTGTCCTGTACATTGCGCC | This work | Construction of plasmid pDL6967 |
| RecA-KO-F1 | AAAAACTGCAGAACGCGGATTTGTCACCTAC | [8] | PCR to check *recA* deletion |
| RecA-KO-R2 | AAAAAGTCGACCGCGGGAAATACCTTTCTG | [8] | PCR to check *recA* deletion |
| recD-KO-F1 | AAAAACTGCAGGTTAATCCGCCAGTTTGACC | [6] | PCR to check *recD* deletion |
| recD-KO-R2 | AAAAAGTCGACGGAGCAGCAAGGTATTCTGG | [6] | PCR to check *recD* deletion |
| RuvA-KO-F1 | AAAAACTGCAGGATCCCGACGTGATTACTCC | [3] | PCR to check *ruvAB* deletion |
| RuvB-KO-R2 | AAAAAGTCGACTGACTGGTGTAGCGATG | [3] | PCR to check *ruvAB* deletion |
| Synth*lacZ*.F | TATCCGCCACCCGCTCTTTC | This work | To generate a 3028 bp fragment to be used as a template for making a Southern probe within the synthetic *lacZ* region |
| Synth*lacZ*.R | CGCCGCTTACAAAACAAGCT | This work |  |

Triple Chi array sequence on the origin proximal side:

GCTGGTGGTCGATGCTGAGCTGGTGGACACGCGCTGGCTGGTGG

Triple Chi array sequence on the origin distal side:

CCACCAGCCGCCATGTGACCACCAGCGAGTCTGCGCCCACCAGC

**References**

1. Khlebnikov, A., Datsenko, K.A., Skaug, T., Wanner, B.L., and Keasling, J.D., Homogeneous expression of the P(BAD) promoter in Escherichia coli by constitutive expression of the low-affinity high-capacity AraE transporter. Microbiology (Reading), 2001. **147**(Pt 12): p. 3241-7.

2. Wertman, K.F., Wyman, A.R., and Botstein, D., Host/vector interactions which affect the viability of recombinant phage lambda clones. Gene, 1986. **49**(2): p. 253-262.

3. Eykelenboom, J.K., Blackwood, J.K., Okely, E., and Leach, D.R., SbcCD causes a double-strand break at a DNA palindrome in the Escherichia coli chromosome. Mol Cell, 2008. **29**(5): p. 644-51.

4. Blattner, F.R., Plunkett, G., 3rd, Bloch, C.A., Perna, N.T., Burland, V., Riley, M., Collado-Vides, J., Glasner, J.D., Rode, C.K., Mayhew, G.F., Gregor, J., Davis, N.W., Kirkpatrick, H.A., Goeden, M.A., Rose, D.J., Mau, B., and Shao, Y., The complete genome sequence of Escherichia coli K-12. Science, 1997. **277**(5331): p. 1453-62.

5. Merlin, C., McAteer, S., and Masters, M., Tools for characterization of Escherichia coli genes of unknown function. J Bacteriol, 2002. **184**(16): p. 4573-81.

6. Darmon, E., Eykelenboom, J.K., Lincker, F., Jones, L.H., White, M., Okely, E., Blackwood, J.K., and Leach, D.R., E. coli SbcCD and RecA control chromosomal rearrangement induced by an interrupted palindrome. Molecular cell, 2010. **39**(1): p. 59-70.

7. White, M.A., Eykelenboom, J.K., Lopez-Vernaza, M.A., Wilson, E., and Leach, D.R., Non-random segregation of sister chromosomes in Escherichia coli. Nature, 2008. **455**(7217): p. 1248-50.

8. Amarh, V., White, M.A., and Leach, D.R.F., Dynamics of RecA-mediated repair of replication-dependent DNA breaks. J Cell Biol, 2018. **217**(7): p. 2299-2307.
