# Supplementary material for: Isolation and purification of DNA double-strand break repair intermediates for understanding complex molecular mechanisms
Source: PLoS One. 2024 Oct 11;19(10):e0308786. doi: 10.1371/journal.pone.0308786 (PMC11469543; doi:10.1371/journal.pone.0308786)
Supplement: S1 Raw images — (PDF) [file pone.0308786.s003.pdf]

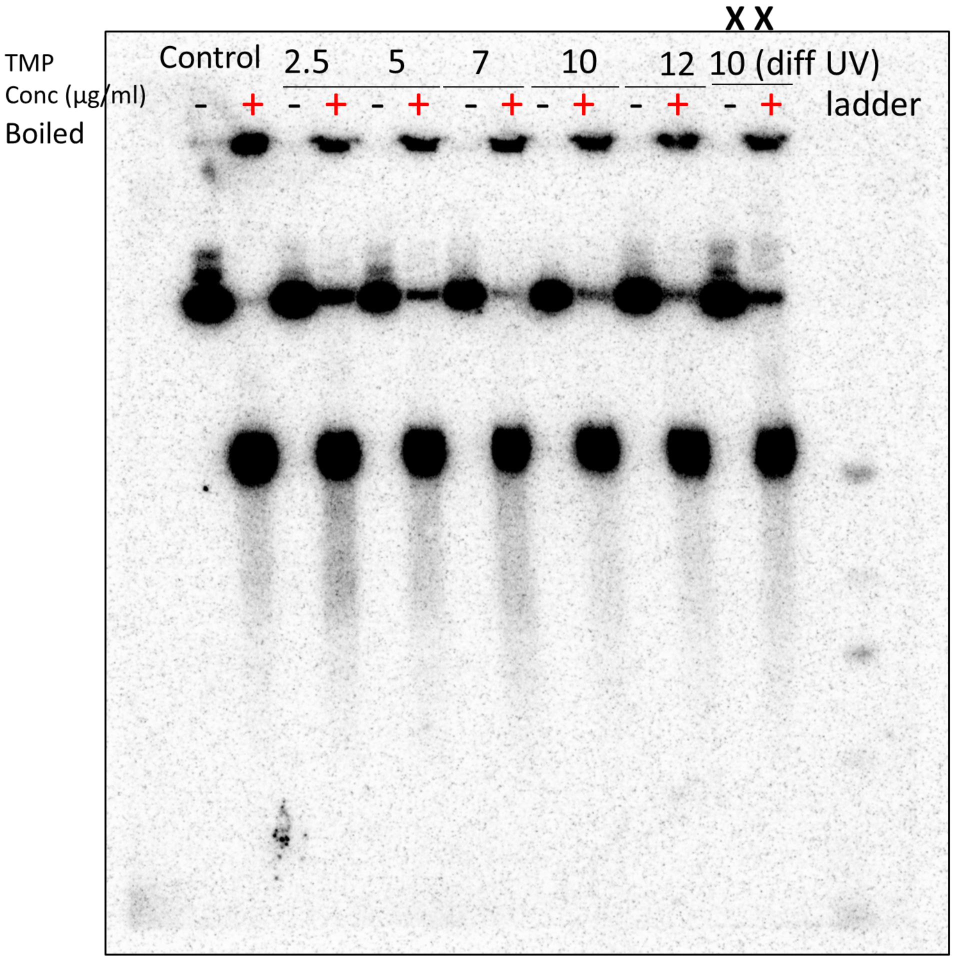
TMP
Conc (µg/ml)
Boiled
Control
2.5
5
7
10
12
10 (diff UV)
X X
- + - + - + - + - + - + - +
ladder

Figure 02

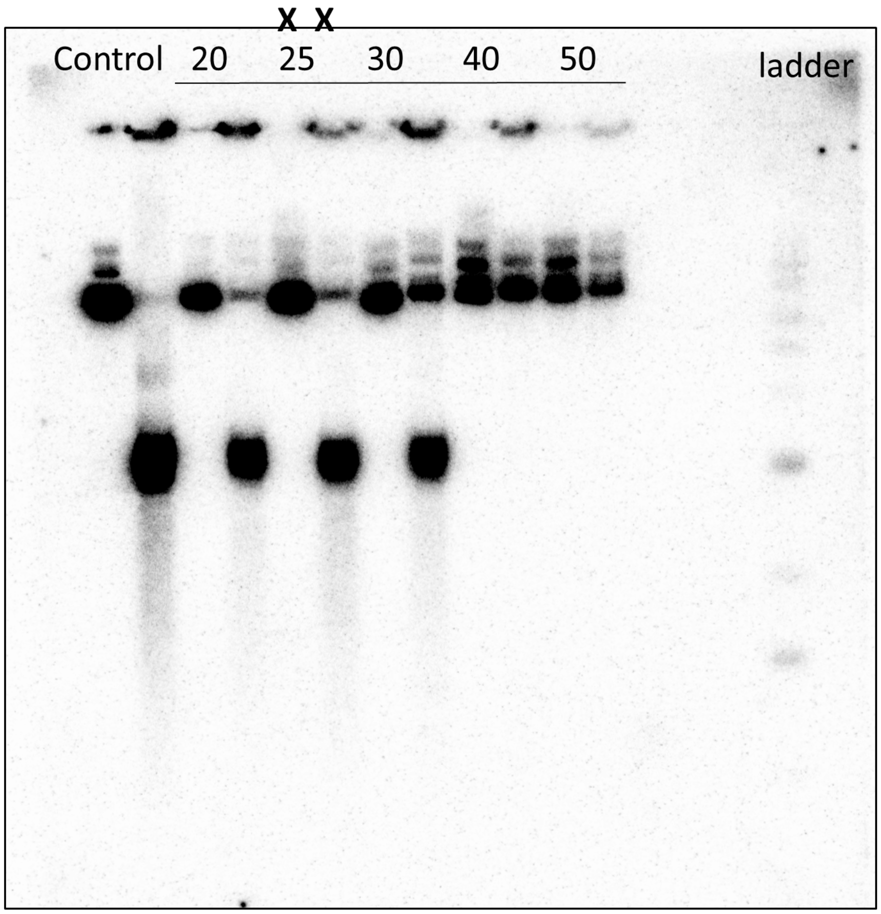
X X
Control
20
25
30
40
50
ladder

1----Undig

2-----26 kb DSB+

3-----26 kb DSB-

4------14 kb DSB+

5------14 kb DSB-

6-------- 16 kb DSB+

7-------16kb DSB-

8------- 8 kb DSB+

9------8 kb DSB-

10-----23 kb DSB+

11-----23 kb DSB-

14------Undig

Figure 4(B)

2-7-------------digested by I-SceI enz

8,9--------------digested by NdeI enz

10,11-----------digested by SalI enz

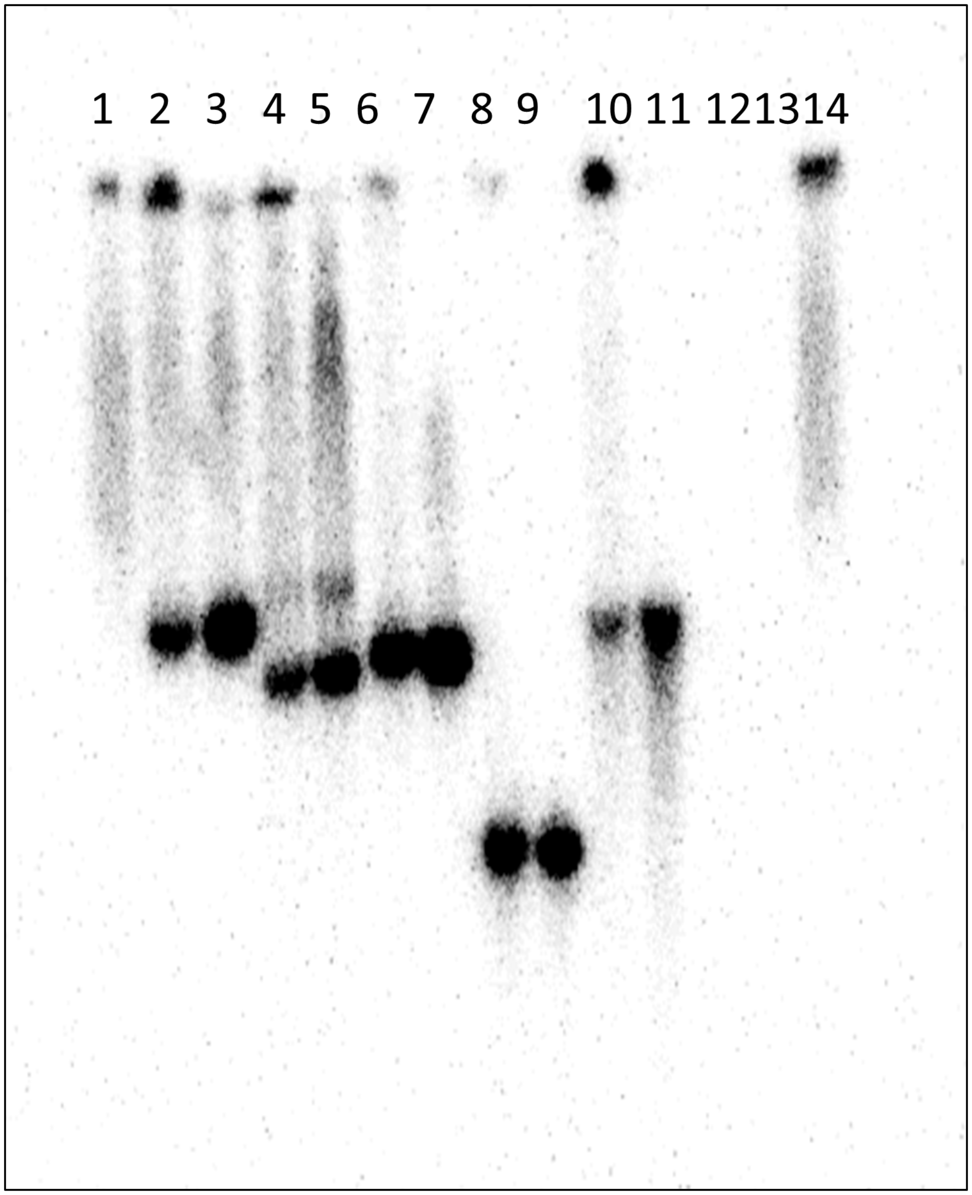

Figure 5(B)

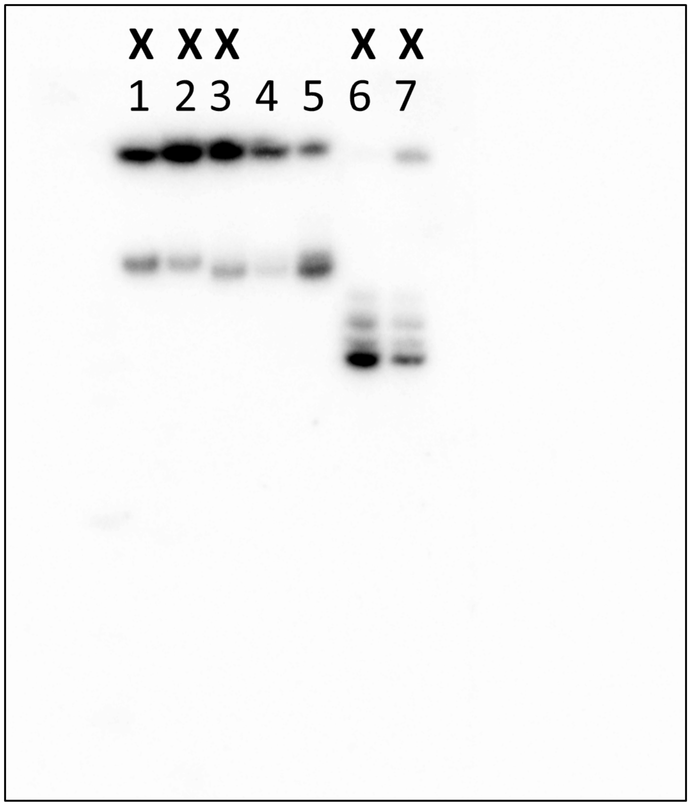

1. Rec+ cells (strain DL4184) Not crosslinked
2. ΔRuvAB cells (strain DL4243), Not crosslinked
3. ΔRuvAB, lacZ: Pal246 (strain DL5743) Not crosslinked
4. **ΔRuvAB, lacZ: Pal246 (strain DL5743) Crosslinked**
5. **ΔRuvAB, lacZ+ (strain DL5744) Crosslinked,**

1-5 lane digested with I-SceI enz

6. ΔRuvAB, lacZ+ (strain DL5744) Not boiled
7. ΔRuvAB, lacZ+ (strain DL5744) Boiled

6-7 lane digested with NdeI

Figure 5(C)

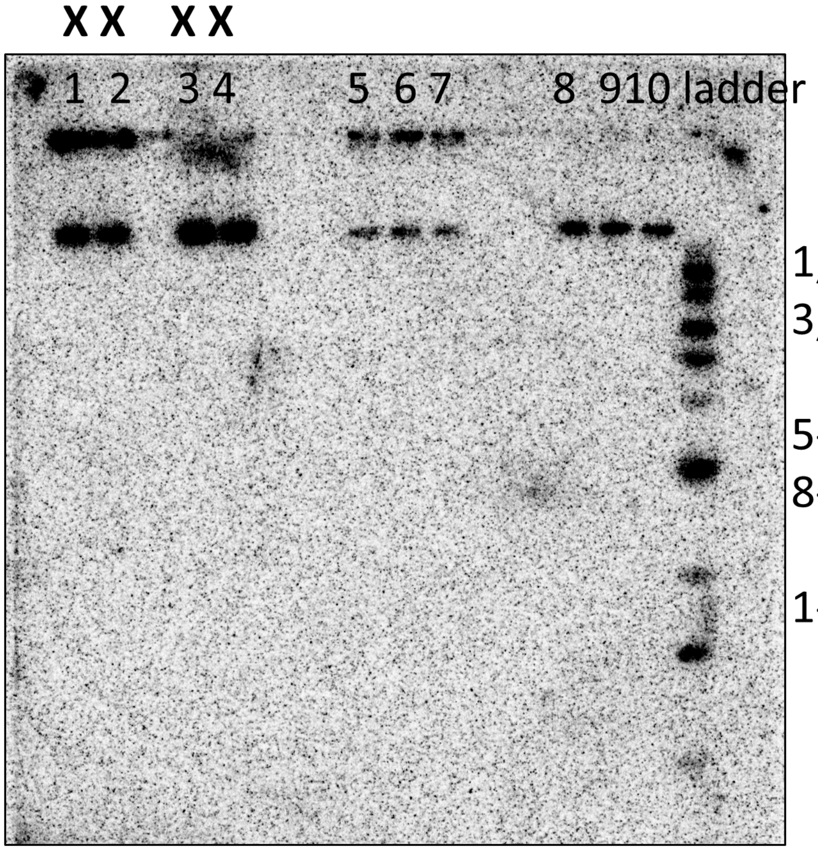

1,2: ΔRuvAB, lacZ: Pal246 (strain DL5743) Not crosslinked
3,4: ΔRuvAB, lacZ+ (strain DL5744) Not Crosslinked

5-7: ΔRuvAB, lacZ: Pal246 (strain DL5743) Crosslinked
8-10: ΔRuvAB, lacZ+ (strain DL5744) Crosslinked

1-10: well content of 1st gel electrophoresis loaded here

Figure 06

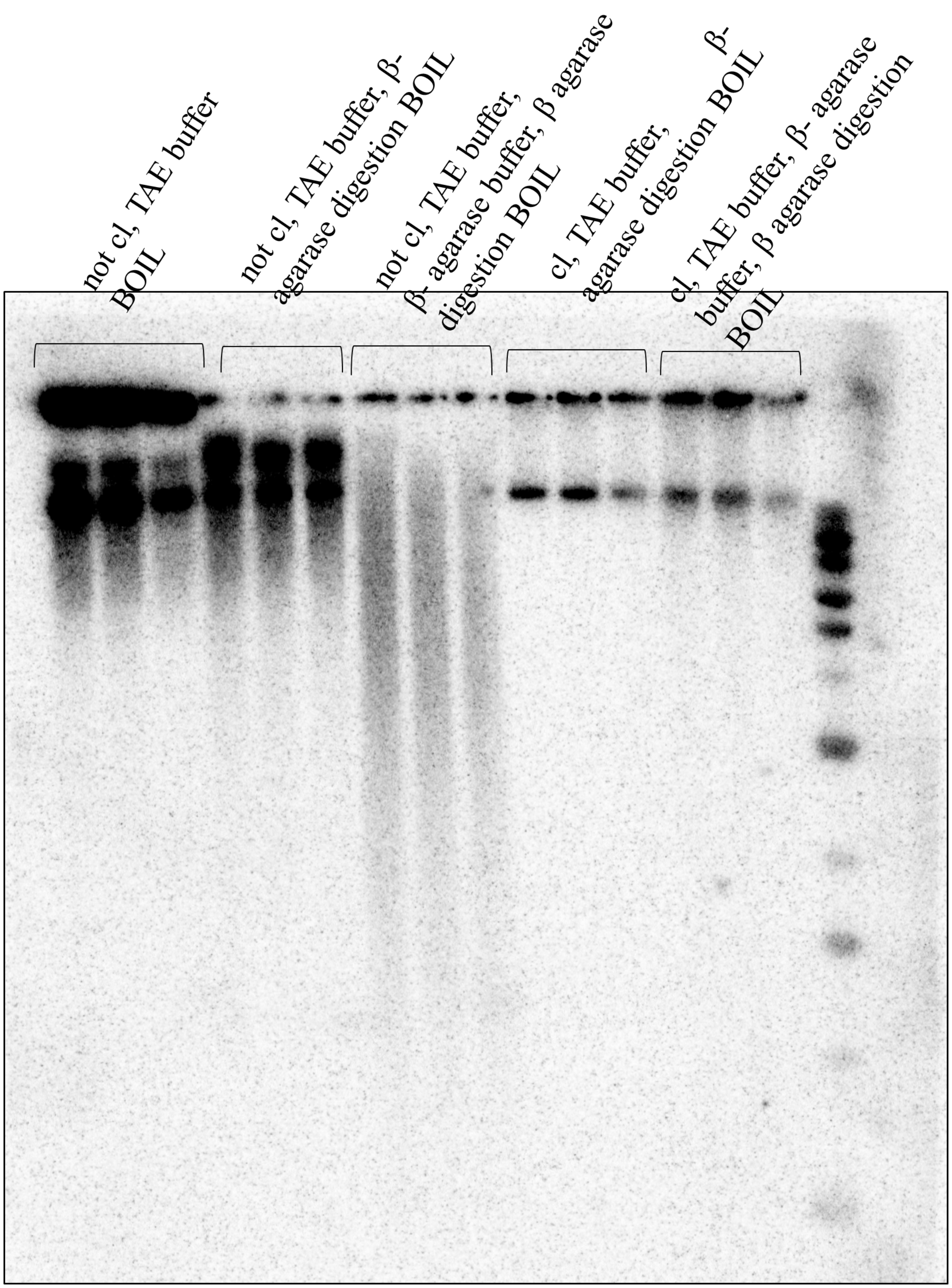

not cl, TAE buffer BOIL
not cl, TAE buffer, β-agarase digestion BOIL
not cl, TAE buffer, β- agarase buffer, β agarase digestion BOIL
cl, TAE buffer, β-agarase digestion BOIL
cl, TAE buffer, β- agarase buffer, β agarase digestion BOIL

Figure 7 (B)

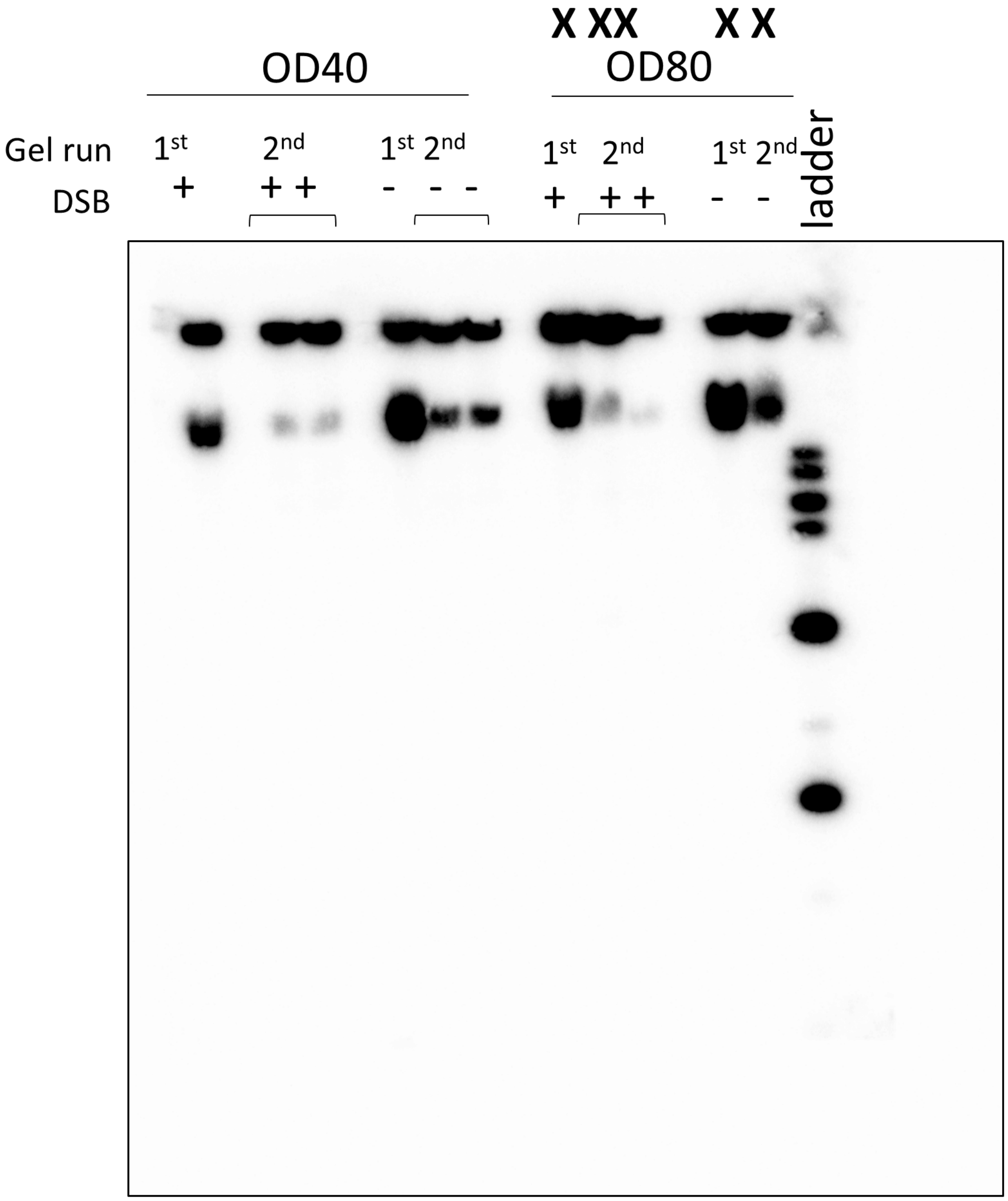
X XX X X
OD40
OD80
Gel run
1st
2nd
1st 2nd
1st
2nd
1st 2nd
DSB
+
+ +
- - -
+
+ +
- -
ladder

Figure 8(A)

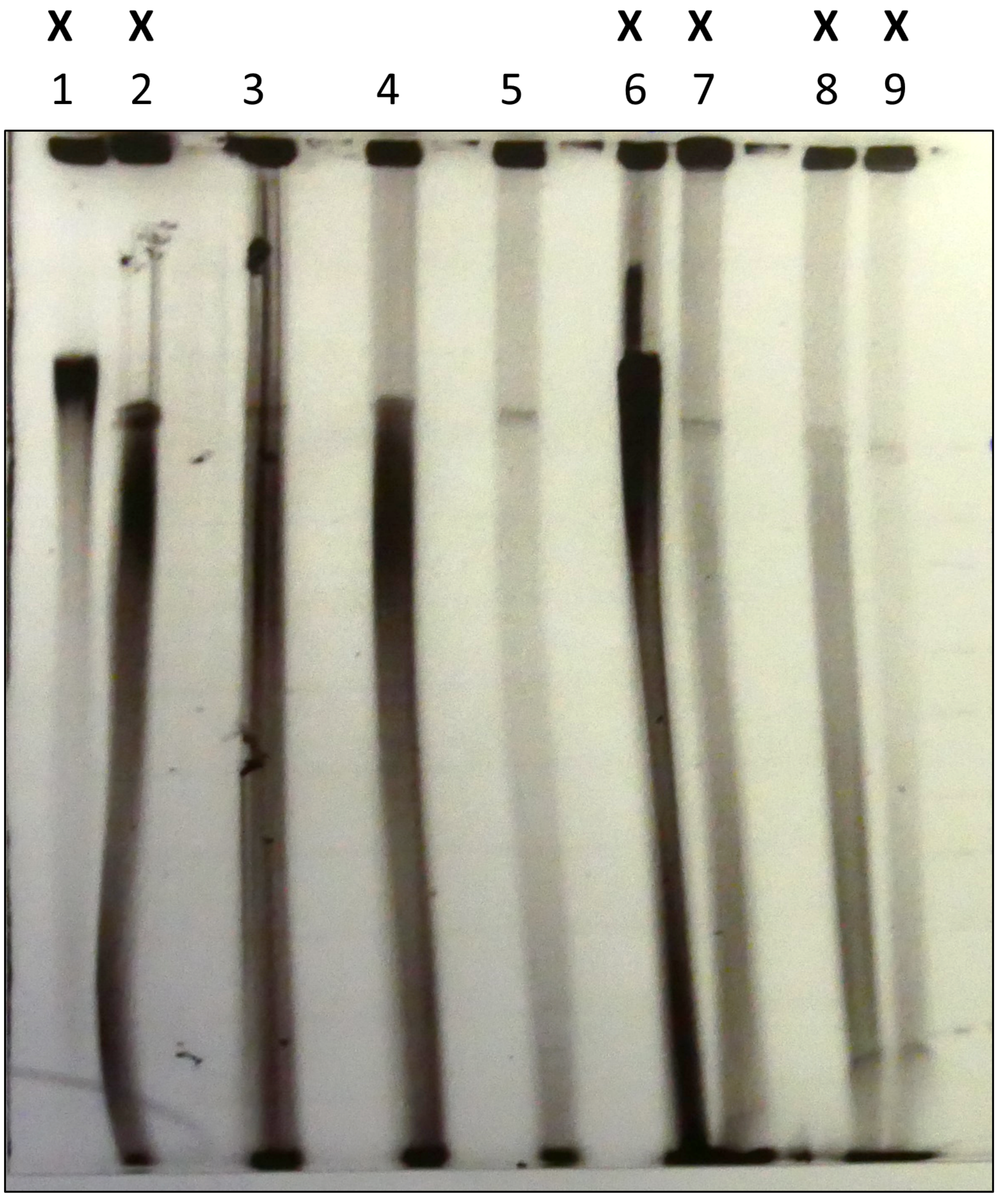

1. Plug made from O/N culture, DSB- cell: undigested
2. Plug made from O/N culture, DSB- cell: digested
3. DSB- cells: digested
4. DSB+ cells: digested with a single enzyme
5. DSB+ cells: digested with double enzymes
6. DSB+ cells: plug made from OD80 cells: digested with a single enzyme
7. DSB+ cells: plug made from OD80 cells: digested with double enzymes

1-7 lane: crosslinked

8. DSB+ cells: Not crosslinked, digested with a single enzyme
9. DSB+ cells: Not crosslinked, digested with double enzyme

1-5 and 8-9: plugs made from OD40 cells

Figure 8(B)

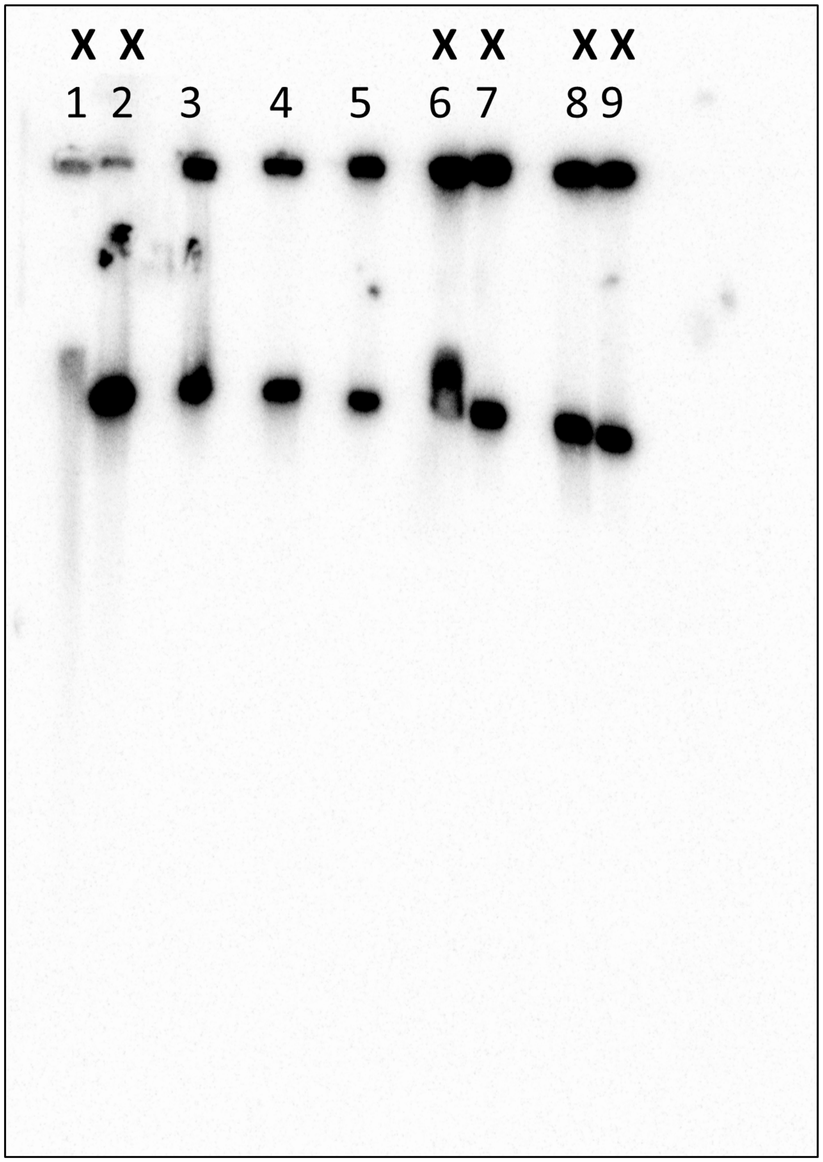

1. Plug made from O/N culture, DSB- cell: undigested
2. Plug made from O/N culture, DSB- cell: digested
3. DSB- cells: digested
4. DSB+ cells: digested with a single enzyme
5. DSB+ cells: digested with double enzymes
6. DSB+ cells: plug made from OD80 cells: digested with a single enzyme
7. DSB+ cells: plug made from OD80 cells: digested with double enzymes

1-7 lane: crosslinked

8. DSB+ cells: Not crosslinked, digested with a single enzyme
9. DSB+ cells: Not crosslinked, digested with double enzyme

Figure 09

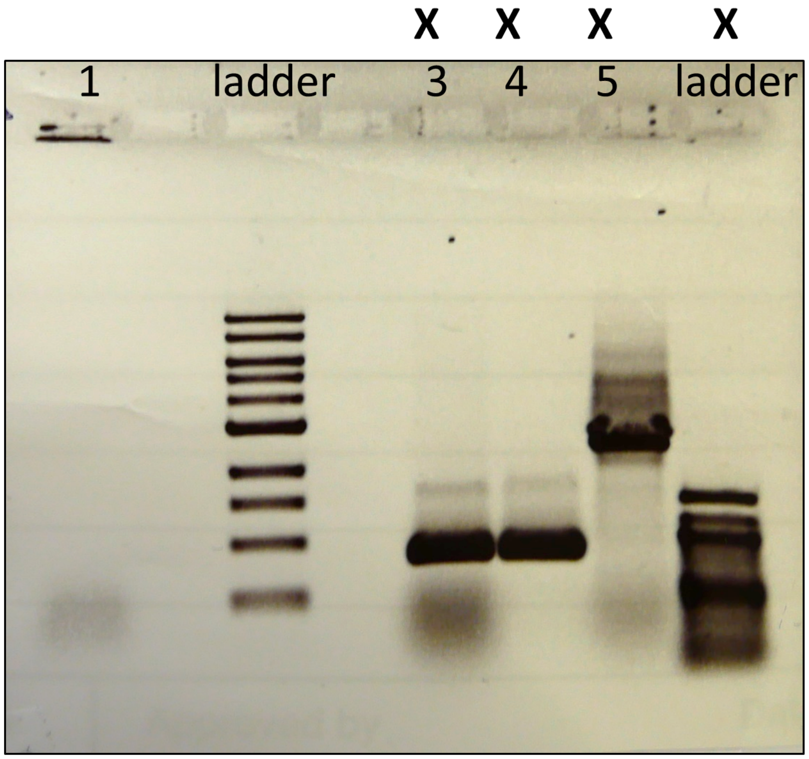

1: DNA sample after two rounds of gel electrophoresis

3, 4, 5: Different experiment (New bacterial strain construction)

3 and 4: checking new strain for RuvAB gene deletion
5: Negative control for RuvAB gene deletion
