# Supplementary material for: Correction: Isolation and purification of DNA double-strand break repair intermediates for understanding complex molecular mechanisms
Source: PLoS One. 2025 Sep 5;20(9):e0331774. doi: 10.1371/journal.pone.0331774 (PMC12412918; doi:10.1371/journal.pone.0331774)
Supplement: S1 File — (PDF) [file pone.0331774.s001.pdf]

RESEARCH ARTICLE

Isolation and purification of DNA double-strand break repair intermediates for understanding complex molecular mechanisms

Tahirah Yasmin[1¤a], **Benura Azeroglu**[1¤b], **Fares Osam Yanez-Cuna**[1¤c], **Sally Jones**[1,2¤d¤e], **Patrick Yizhi Cai**[1,2¤e], **David R. F. Leach**[1]*

1 Institute of Cell Biology, University of Edinburgh, Edinburgh, United Kingdom, **2** Manchester Institute of Biotechnology, University of Manchester, Manchester, United Kingdom

¤a Current address: Department of Biochemistry and Molecular Biology, University of Dhaka, Dhaka, Bangladesh
¤b Current address: Laboratory of Genome Integrity, National Cancer Institute, NIH, Bethesda, Maryland, United States of America
¤c Current address: Laboratory of Spatial Regulation of Genomes, Department of Genomes and Genetics, Institut Pasteur, Paris, France
¤d Current address: Department of Cell and Developmental Biology, John Innes Centre, Norwich Research Park, Norwich, United Kingdom
¤e Current address: Manchester Institute of Biotechnology, University of Manchester, Manchester, United Kingdom
* D.Leach@ed.ac.uk

Check for updates

OPEN ACCESS

Citation: Yasmin T, Azeroglu B, Yanez-Cuna FO, Jones S, Cai PY, Leach DRF (2024) Isolation and purification of DNA double-strand break repair intermediates for understanding complex molecular mechanisms. PLoS ONE 19(10): e0308786. https://doi.org/10.1371/journal.pone.0308786

Editor: Hari S. Misra, Gandhi Insititute of Technology and Management, INDIA

Received: January 21, 2024

Accepted: July 31, 2024

Published: October 11, 2024

Copyright: © 2024 Yasmin et al. This is an open access article distributed under the terms of the Creative Commons Attribution License, which permits unrestricted use, distribution, and reproduction in any medium, provided the original author and source are credited.

Data Availability Statement: All relevant data are within the manuscript and its Supporting Information files.

Funding: DRFL MR/M019160/1 Medical Research Council (UK) https://mrc.ukri.org TY EMBO short term fellowship 7879 https://www.embo.org The sponsors played no role in the study design, data collection and analysis, decision to publish, or preparation of the manuscript.

Abstract

Branched DNA molecules are key intermediates in the molecular pathways of DNA replication, repair and recombination. Understanding their structural details, therefore, helps to envisage the mechanisms underlying these processes. While the configurations of DNA molecules can be effectively analysed in bulk using gel electrophoresis techniques, direct visualization provides a complementary single-molecule approach to investigating branched DNA structures. However, for microscopic examination, the sample needs to be free from impurities that could obscure the molecules of interest, and free from the bulk of unwanted non-specific DNA molecules that would otherwise dominate the field of view. Additionally, in the case of recombination intermediates, the length of the DNA molecules becomes an important factor to consider since the structures can be spread over a large distance on the chromosome *in vivo*. As a result, apart from sample purity, efficient isolation of large-sized DNA fragments without damaging their branched structures is crucial for further analysis. These factors are illustrated by the example of DNA double-strand break repair in the bacterium *E. coli*. In *E. coli* recombination intermediates may be spread over a distance of 40 kb which constitutes less than 1% of the 4.6 Mb genome. This study reveals ways to overcome some of the technical challenges that are associated with the isolation and purification of large and complex branched DNA structures using *E. coli* DNA double-strand break repair intermediates. High-molecular weight and branched DNA molecules do not run into agarose gels subjected to electrophoresis. However, they can be extracted from the wells of the gels if they are agarose embedded, by using β-agarase digestion, filtration, and concentration.

Competing interests: The authors have declared that no competing interests exist.

Furthermore, a second round of gel electrophoresis followed by purification is recommended to enhance the purity of the specific DNA samples. These preliminary findings may prove to be pioneering for various single-molecule analyses of large and complex DNA molecules of DNA replication, repair and recombination.

1. Introduction

For a cell to survive and for the integrity of its genome, DNA double-strand breaks (DSBs) need to be correctly repaired. A faithful system of DSB repair prevents genome rearrangements that may result in the accumulation of mutations that may lead to the evolution of bacterial resistance to antibiotics and in the case of multicellular organism cancer. At every cycle of DNA replication, the cell risks the generation of DSBs and their frequency scales approximately with genome size. Accordingly, *Escherichia coli* cells experience one break every five replication cycles [1] while human cells experience about fifty DSBs during one round of DNA replication [2]. To repair these breaks, *E. coli* uses homologous recombination (HR), where the undamaged sister chromosome is used as a template. It is anticipated that DNA intermediates in this process will be branched molecules, such as repair forks, D-loops, and Holliday junctions (HJs). Despite an excellent appreciation of the biochemistry of individual purified proteins, the mode and scope of their *in vivo* actions are difficult to ascertain because of the short-lived nature of these intermediates, the overlapping actions of the pathway proteins and unknown aspects of the *in vivo* environment. Studying the composition and distribution of the branched repair intermediates, which can be seen in recombination mutants, is, therefore, crucial for achieving this goal.

Pulsed-field gel electrophoresis and two-dimensional gel electrophoresis followed by Southern hybridization are the techniques of choice that have been applied to study the branched structures of DSB repair *in vivo*. However, while pulsed-field gel electrophoresis is effective at resolving long linear DNA molecules, it is unable to differentiate between the various branched structures formed, such as 3-way (i.e., D-loops, replication forks) or 4-way junctions (i.e. Holliday junctions) which are readily trapped in the wells [3]. In contrast, native-native two-dimensional gel electrophoresis is extremely helpful for identifying 3-way and 4-way DNA structures in molecules under approximately 10 kb, but it is not applicable to longer molecules. Furthermore, structures with an equal number of branches exhibit similar migration patterns in the gel. As a result, this technique fails to distinguish clearly converged replication forks, Holliday junctions, and hemicatenanes, all of which have four DNA branches. Additionally, the approach is unable to distinguish D-loops from replication forks [4], as both of them have three DNA branches. However, all these molecules are central to different molecular mechanisms including DNA repair, replication and recombination.

By contrast, direct visualization methods can be utilized to investigate longer DNA molecules and to separate complicated branched structures from one another. For instance, using transmission electron microscopy (TEM), it should be possible to detect whether double Holliday junctions are present in the 20–40 kb area surrounding a DSB site. It should also be useful to assess whether replication forks are present in the repair intermediates, which will aid to narrow down the distance at which the repair synthesis can begin from the DSB. TEM also makes it possible to distinguish between single-stranded DNA (ssDNA) and double-stranded DNA (dsDNA), with the former appearing as a fiber that is 10 nm thick and the latter having a decreased thickness of 5–7 nm in naked DNA spreads [5].

TEM has never been carried out in relation to a DSB generated at a specific genomic location, which would allow greater sophistication of investigation of the structures than has previously been possible, given knowledge of the precise location of the initiating DSB and any other DNA features present at that genomic site.

However, a major challenge for a successful microscopic investigation or any other extensive molecular analysis that involve the examination of a single large and branched DNA molecule is to prepare a pure sample of such a DNA fragment that constitutes a small (<1%) proportion of the total genomic DNA without shearing and without significant movement of branches during the isolation procedure. This study has focused on optimizing a protocol for preparing a branched DNA sample containing DNA recombination intermediates that can be extensively analyzed for different purposes. Two different analysis strategies have been investigated here. In both strategies, DSBs were generated at a specific location using an inducible SbcCD-palindrome system that generates a replication-dependent DSB in close to 100% of *E. coli* genomes [6]. In the first strategy, the DNA region surrounding the DSB site was isolated by cleavage of the chromosome with the rare cutting nuclease I-SceI at two sites specifically engineered in the genome. In the second, the DSB site was embedded in a synthetic region of DNA devoid of 4-base recognition sites for restriction endonucleases (REs) that were used to fragment the rest of the chromosome. In conclusion, our preferred protocol comprises the following steps:

1. Generating a replication-dependent DSB using an inducible SbcCD-palindrome system.
2. Placing arrays of the recombination initiation site Chi on each side of the DSB site with no other Chi sites in the immediate vicinity, to ensure that intermediates formed in the region analyzed are generated in relation to known DNA sequences at known locations.
3. Genetically trapping branched molecules by blocking the migration and resolution of Holliday junctions in a *ruvAB* mutant. These strains are unable to catalytically branch migrate and resolve HJs, which as a result are accumulated *in vivo* during DSB repair of a replication-dependent break [3].
4. Physically trapping DNA structures *in vivo*, prior to DNA extraction, by crosslinking DNA using psoralen and UV light.
5. Ensuring that the 40 kb surrounding the DSB site is composed of DNA devoid of 4-bp palindromic targets for restriction endonucleases that can be used to fragment the rest of the chromosome for removal during purification.
6. Purifying the branched DNA of interest from the small restriction endonuclease fragments by agarose gel electrophoresis.
7. Extracting the branched DNA from the agarose gel.
8. Purifying the branched DNA from contaminants that would impede direct visualization.
9. Preparing the final sample.

2. Materials and methods

2.1 Bacterial strains, plasmids and oligonucleotides

All strains used are listed in Supplementary Information S1 Table. The strains were derived from BW27784, which has been designed to ensure efficient and uniform induction of gene expression by arabinose and in which arabinose is not used as a carbon source [7]. The

plasmids used in the construction of the strains and the oligonucleotides used in the construction of the plasmids are listed in Supplementary Information S1 Table.

2.2 Isolation of chromosomal DNA in agarose plugs and psoralen crosslinking of the DNA

DNA isolation in agarose plugs, psoralen crosslinking of the DNA and restriction enzyme digestion of the agarose-embedded DNA were carried out as described in [8]. Unless otherwise indicated, overnight bacterial cultures grown in 5 ml L-broth were diluted to an OD_{600nm} of 0.02 in LB medium supplemented with 0.5% glucose. The cells were allowed to grow at 37˚C to an OD_{600nm} of 0.2–0.3 and then re-diluted in LB with 0.5% glucose to an OD_{600nm} of 0.02. They were divided into two sub-cultures and left to grow for another 15 minutes. After that, 0.2% arabinose was added to one sub-culture in order to induce DSB formation mediated by SbcCD. An hour after the addition of arabinose, cells were harvested at 4˚C and washed 3 times in TEN buffer (50 mM Tris, 50 mM EDTA, 100 mM NaCl, pH 8.0). After the final wash, cells were re-suspended in the calculated volume of TEN buffer to give a total cell OD_{600nm} of 40 and mixed with an equal volume of melted 0.8% (w/v) low melting point (LMP) agarose in distilled H_2O. Immediately, the mixture was poured into plug moulds. Once set at 4˚C, the plugs were incubated overnight at 37˚C in proteinase K solution (1 mg/ml) dissolved in NDS buffer (0.5 M EDTA, 10 mM Tris, 0.55 M NaOH, 36.8 mM lauroyl sarcosine; pH 8.0; 1 ml/plug). On the next day, the solution was replaced with fresh proteinase K solution for another overnight incubation in NDS buffer. Finally, the plugs were stored at 4˚C in fresh NDS buffer without proteinase K.

For crosslinking, after the cells were harvested at 4˚C, the pellets were suspended in 1.6 ml of ice-cold PBS (137 mM NaCl, 2.7 mM KCl, 10 mM Na_2HPO_4 and 1.8 mM KH_2PO_4) and 0.4 ml of trimethylpsoralen (TMP) stock solution (200 μg/ml TMP in 100% ethanol) was added to each cell suspension to a final concentration of 40 μg/ml. After addition of TMP, the cells were incubated for 10 minutes in the dark on ice. Afterwards, the cells were placed on precooled glass petri dishes, inside a UVP crosslinker on a precooled metal surface and irradiated at UV exposure setting of 200,000 μjoules/cm^2. Following the irradiation, the cell suspensions were transferred back to Eppendorf tubes on ice and spun down. The pellets were then used for DNA extraction in agarose plugs as described above.

Before digestion of the DNA, a plug was washed in 1 ml of TE buffer for 5 hours, changing the buffer every hour. Before the 6th wash, each plug was divided into two halves with a clean scalpel. For the 6th wash, instead of TE buffer, 1 ml of 1X appropriate restriction buffer was used per plug and this wash took place at 37˚C for 5 hours under gentle agitation. Once washed, the plugs were digested in 1 ml of fresh 1X appropriate restriction buffer with 100 U of enzyme per plug. Digestions were set overnight at 37˚C under gentle rocking. Next day the digestion mixture was replaced with fresh buffer and fresh enzyme (100 U) and the reaction was carried out for another three hours.

2.3 Agarose gel electrophoresis

DNA fragments resulting from the digestion of *E. coli* genomic DNA were separated on 0.5% (w/v) MELFORD agarose electrophoresis grade gels. The appropriate amount of agarose was melted in 1X TBE (89 mM Tris-borate, 2 mM EDTA) and allowed to cool to 55˚C before being poured. Gels were run at 38–40 V for up to 18–22 hours. Afterwards the gels were submerged in an ethidium bromide staining solution (4 μl of EtBr stock per 100 ml of distilled water) and washed for 30 minutes under agitation. The staining tray was wrapped in aluminium foil during washing. Finally, the DNA was visualised using the GelDoc XR+ system (BioRad).

2.4 Radioactive detection of DNA

DNA fragments were detected using $^{32}P\alpha$-dATP incorporated radio-labelled DNA probes (prepared using Stratagene Prime-It II random primer labelling kit according to the manufacturer's instructions). Probes were hybridized to membranes overnight at 65˚C in 10-15ml of hybridization buffer (7% SDS (Sodium dodecyl sulfate), 0.5 M NaH_2PO_4, 1 mM EDTA). Membranes were washed for 15 minutes at 60˚C in 2X SSC (1X SSC: 0.15 M NaCl, 0.015 M Na-citrate) supplemented with 0.1% SDS and then 30 minutes in 0.5X SSC supplemented with 0.1% SDS.

2.5 β-agarase digestion of agarose gel to extract large chromosomal DNA fragments and clean-up and concentration of β-agarase treated DNA

The optimum temperature for β-agarase activity is 40–45˚C. Therefore, only low melting point agarose is suitable for β-agarase digestion as the solution must be liquid at the incubation temperature of 42˚C. In this procedure, the DNA-containing 0.5% (W/V) low melting point agarose (UltraPure Low Melting Point Agarose, Catalogue No- 16520050) gel slice was first cut out from the gel using a clean, sterile scalpel. Afterwards the gel was equilibrated by washing the gel slice in a 15 ml falcon tube with 15 ml of 1X TAE Buffer (2 M Tris-base, 0.95 M Glacial acetic acid and 0.5 M EDTA, made with Milli-Q water) at room temperature for minimum 3 hours under mild agitation. After the wash, the buffer was removed, and the gel slice was transferred to a 1.5 ml Eppendorf tube. Then, the agarose was melted by incubation at 60˚C for 5 minutes. Immediately after, the molten agarose was cooled to 42˚C in another water bath and 2 units of β-agarase were added to each sample. The reaction was left at 42–43˚C for 2 hours. The digested agarose containing DNA solution was next run through a GE Healthcare Illustra Microspin™ G-25 Column according to manufacturer's instructions to remove the carbohydrates and any undigested agarose. Afterwards, the solution was concentrated using a Microcon DNA Fast Flow Centrifugal Filter Unit (Catalogue No: MRCF0R100) according to the manufacturer's instructions and reduced from a 120–150 μl volume to a final volume of 20–30 μl. Finally, the DNA concentration of the sample was measured using Qubit dsDNA High-sensitivity assay by following the manufacturer's guidelines.

2.6 Statistical analyses

The DNA was quantified from the radiolabelled membranes using ImageQuant software. The significance of the mean difference was analysed by paired t-test.

3. Results

3.1 Generating and preserving branched double-strand break repair (DSBR) intermediates from a single genomic DSB site

A replication-dependent DSB system that permits the introduction of a site-specific DSB on one of a pair of replicating sister chromosomes was developed by the Leach laboratory [6]. In this system, an inducible structure-specific endonuclease, SbcCD, recognizes and cleaves a DNA hairpin that forms on the lagging-strand template following the replication of a palindromic sequence (Fig 1). As a result, a DSB is generated on only one of the sister chromosomes, which is subsequently repaired using the intact homologous sister chromosome as a template. Despite frequent cleavage (likely to occur at every replication cycle) resulting in the death of recombination-deficient strains [6], the repair is rapid and efficient in recombination-proficient strains [9]. These observations suggest frequent and efficient DSBR using

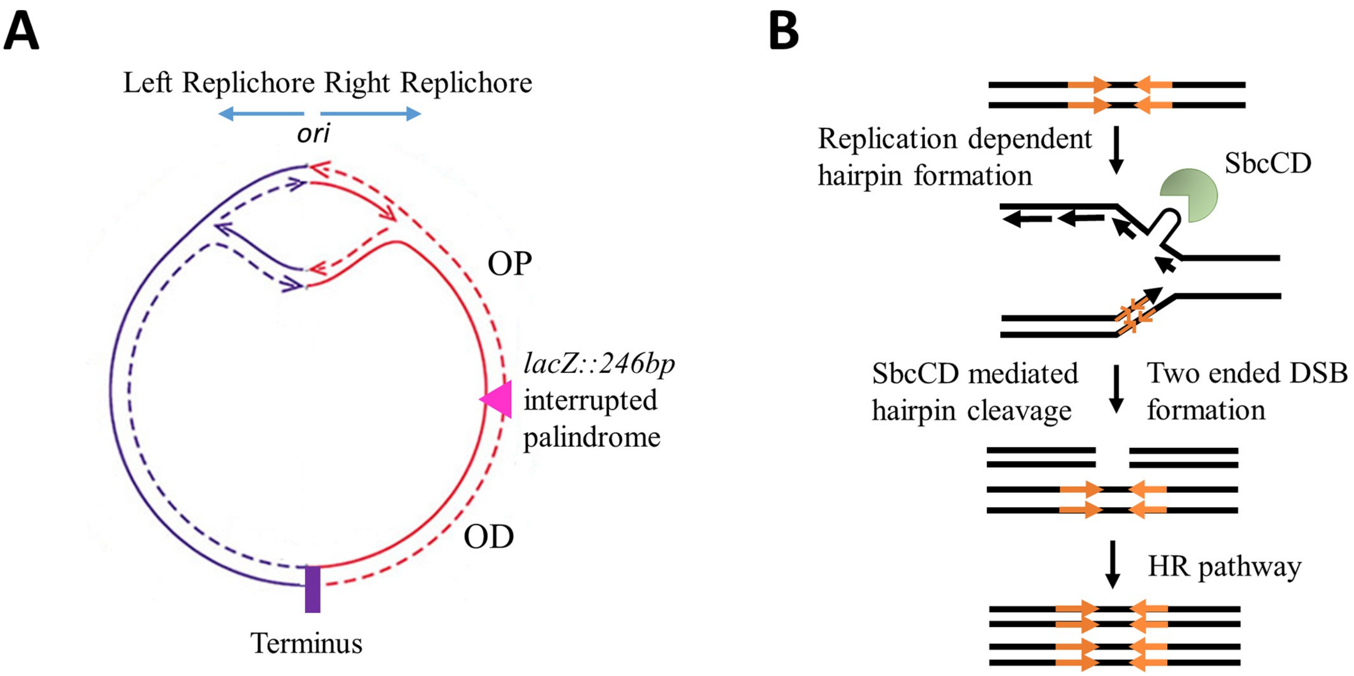

Fig 1. SbcCD/palindrome-induced replication-dependent site-specific DNA double-strand break system in *E. coli*. A) A schematic representation of *E. coli* circular chromosome showing bidirectional replication originating from oriC and the location of the palindrome within the *lacZ* region in the right replichore. B) An illustration showing SbcCD-mediated cleavage of the 246bp interrupted palindrome that results in a two-ended DSB. The palindrome is highlighted by orange arrows.

https://doi.org/10.1371/journal.pone.0308786.g001

homologous recombination, making this system well-suited for the accumulation of site-specific DSBR intermediates in appropriate recombination defective mutants.

DSBR intermediates such as Holiday junctions (HJs) can move along DNA, driven either by enzymatic or spontaneous branch migration, interfering with their capture at the site of their generation. Therefore, enzymatic branch migration activity was restricted using Δ*ruvAB* mutant strains. Spontaneous branch migration of HJs was restricted by *in vivo* DNA crosslinking with TMP prior to DNA isolation. However, too many crosslinks were expected to decrease the digestion efficiency of different restriction enzymes in function of the sequence of their target site. Therefore, to determine the minimal effective psoralen concentration to generate crosslinks within a 7.9 kb DNA fragment at the DSB site, cultures (with no DSB induction) were incubated with different concentrations of psoralen (from 0 to 50 μg/ml) followed by DNA extraction and digestion with NdeI restriction enzyme that would generate the 7.9 kb fragment. After digestion one DNA sample from each psoralen concentration was denatured by incubating the sample at 100˚C, followed by a quick cooling to 4˚C, while another was kept as it was at room temperature. Then all samples were run on an 1% agarose gel and the DSB site-containing fragment was revealed by Southern blotting and hybridization. DNA fragments containing crosslinks will rapidly renature to double strands upon cooling down and therefore will run similarly to their non-denatured DNA molecules on the gel whereas non cross-linked fragments will remain single stranded and run with higher mobility compared to their non-denatured equivalent fragment on the gel and form a band at a position corresponding to 3 kb non-denatured DNA fragments. This would allow one to find out at which TMP concentrations all the 7.9 kb fragments get stabilised by crosslinking. A DNA fragment of 7.9 kb length being stabilised by trimethylpsoralen crosslinking means that there is at least one crosslink every 7.9 kb DNA length which permits rapid renaturation of the DNA double

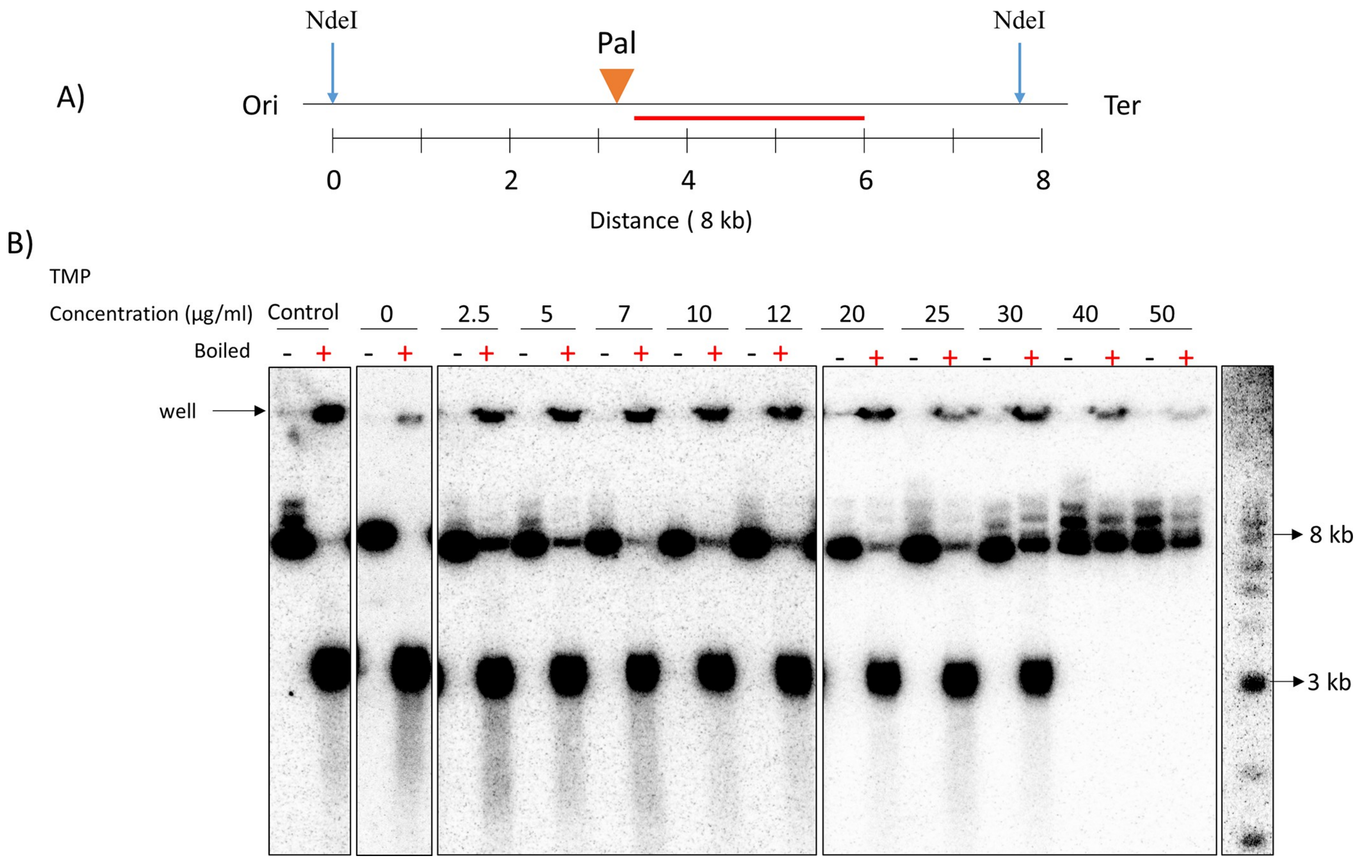

Fig 2. Optimisation of psoralen crosslinking using different concentrations of psoralen. A) NdeI digestion map of the region surrounding the DSB. NdeI restriction sites and the distance between them are marked with blue vertical arrows and numbers (in kb), respectively. The palindrome and the radiolabelled probe that was used to detect the fragment are marked by an orange triangle and a red line, respectively. Ori and Ter mark the orientation of the DNA fragment with respect to the origin and terminus of DNA replication. B) Detection of DNA fragments by Southern blotting and hybridization of samples treated with different concentrations of psoralen (0–50 μg/ml). '+' indicates that the sample was boiled and immediately cooled and '-' indicates that the sample was not boiled. 1 kb NEB ladder was used as a marker. The control DNA samples were neither treated with TMP nor irradiated.

https://doi.org/10.1371/journal.pone.0308786.g002

strands together and this was observed for psoralen concentrations of 40 μg/ml and above (Fig 2). Some interference with NdeI digestion was observed as a function of increasing psoralen concentration in the form of higher-than-expected bands. DNA could also be detected in the wells of the boiled samples regardless of presence of TMP which shows that after being boiled and immediately cooled down, some DNA become trapped in the wells. This experiment showed that only from a concentration of 40.0 μg/ml of TMP was the band corresponding to the ssDNA fragment completely undetectable, which indicates that all of the 7.9 kb fragments were being crosslinked at least once.

3.2 Separating large DNA fragments containing branched DSBR intermediates from the rest of the chromosome

Two different approaches were taken here. Both approaches involve enzymatic digestion and are illustrated in Fig 2. In one strategy (Fig 3A), an *E. coli* strain harboring two cutting sites for the homing endonuclease I-SceI surrounding the DSB site, 10 kb on the origin proximal end and 16 kb on the origin distal end, was constructed. The 18 bp I-SceI target site (`TAGGGATAACAGGGTAAT`) is not naturally present in the *E. coli* genome. I-SceI digestion of the strain therefore produced two DNA fragments: one extremely big fragment representing

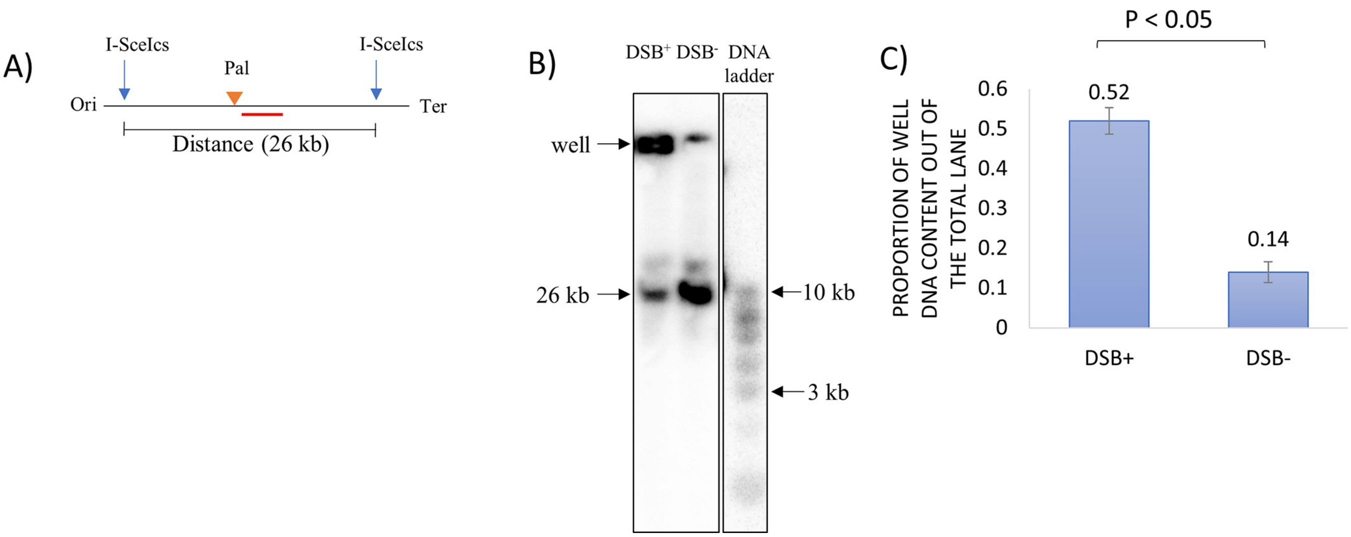

Fig 3. Separating DNA fragments of interest from the rest of the chromosome. A) I-SceI digestion of the chromosome will produce two DNA fragments: an extremely big fragment and a much smaller segment containing the DSB region (palindrome shown by an orange triangle). B) Using a synthetic region devoid of 4-base cutting sites of REs, when digested by the frequent cutters, the rest of the chromosome will be cleaved into small fragments, leaving the 40 kb DSB region (palindrome) intact. C) Design of the 40 kb synthetic DNA region. The palindrome, Chi arrays, and kanamycin resistance gene are indicated by the orange triangle, orange angle bracket and a blue box, respectively. Different restriction sites are marked with black vertical lines and different coloured vertical arrows. The 4 base cutters (BstUI and HhaI) that have been removed from the synthetic region and been used in this study are shown in the homology arms. The selective restriction sites that are kept in the synthetic DNA are shown below the sequence. The 5 kb homology arms to *E. coli* chromosome are indicated by green boxes.

https://doi.org/10.1371/journal.pone.0308786.g003

the remainder of the linearized chromosome, and one large (around 26 kb in length) but much smaller segment representing the DSB region.

The alternative strategy for isolating the DSB region (Fig 3B) involves separating it from the remainder of the chromosome, which was digested into small DNA fragments while the DSB region remained intact. For this purpose, a region of 40 kb including the DSB site in the middle was designed *in silico*, synthesized, and used to replace a natural 40 kb segment of the *E. coli* genome. The synthetic sequence was modified to be devoid of target sites of a set of specific 4-base cutter restriction enzymes. As a result, the rest of the chromosome could be digested into small fragments of around 2–3 kb using these 4 base cutters that would not cut the synthetic region, leaving the 40 kb DSB region intact (Fig 3C). The construction and insertion of the synthetic DNA are described in Supplementary Information S1 File: Construction and integration of a 40 kb synthetic region of DNA.

Once digested, the fragments of interest needed to be collected for further analysis. Therefore, the fragments resulting from the restriction enzyme digestion were electrophoresed in agarose gels to determine their migration properties.

In initial experiments, DNA from a *ruvAB* mutant with a 246 bp palindrome and an inducible SbcCD was digested by I-SceI. The DNA of branched recombination intermediates generated in response to a DSB within a 26 kb fragment from this strain has been predicted to run into a conventional agarose gel whereas the rest of the chromosomal DNA would be trapped in the well due to its very large size. Previous work showed that branched DNA molecules generated within fragments of 6.4 kb in length were able to enter conventional agarose gels but that branched DNA molecules from fragments of 23.7 kb in length were unable to enter pulsed-field gels [3]. To test whether the 26 kb DSB-containing fragment could be isolated in a conventional gel electrophoresis, DNA was collected from two different conditions. In one condition, the production of a DSB at the site-specific locus was induced in the *E. coli*

chromosome and in the other condition, no DSB was formed. These two conditions were used to identify the recombination intermediates that would be generated in response to the DSB. Besides, different DNA fragments with varied lengths of 14 kb, 16 kb, 8 kb and 23 kb from these two conditions were also run in the same gel. Following Southern blotting and probing for the DSB region, it was detected that the majority of the DNA isolated from the strain in which DSB formation was not induced ran on the gel upto various positions reflecting their sizes, while the majority of the DNA isolated from the strain in which DSB formation was induced did not enter the gel and remained in the well (Fig 4). There was a significant difference between the proportion of the well DNA content between the DSB+ and the DSB- strains.

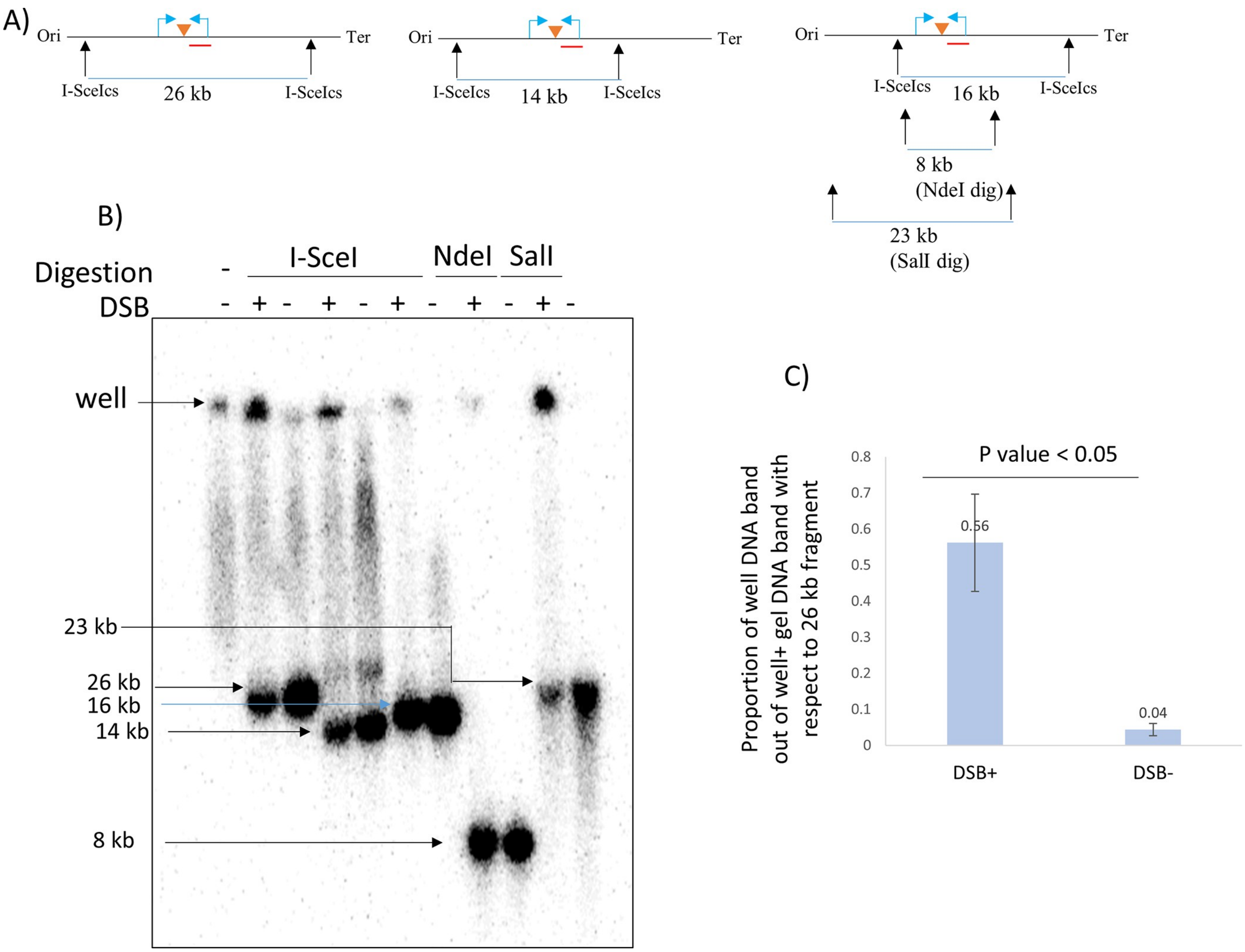

Fig 4. Gel electrophoresis analysis of I-SceI digested DNA fragments. A): I-SceI, NdeI and SalI digestion maps of the *E. coli* chromosomal region surrounding the DSB site. The restriction sites and the distance between them are marked with blue vertical arrows and numbers (in kb), respectively. The DSB site is marked by an orange triangle. Ori and Ter mean origin and terminus, respectively. The radiolabelled probe that was used to detect the fragment is marked by a red line. B): Southern blot of gel electrophoresis of a DSB^+ and a DSB^- strain where a radioactive probe is used to detect a 26 kb, 14 kb and 16 kb DNA fragments surrounding the DSB site, resulting from I-SceI digestion, a 8 kb DNA fragment resulting from NdeI digestion and a 23 kb DNA fragment resulting from SalI digestion. The position of the well and the linear fragments are shown by horizontal black arrows. 1 kb NEB ladder was used as a marker. C): Quantification of branched DNA molecules represented as the proportion of well DNA content out of the sum of well DNA band and gel DNA band. The values are shown on the top of each bar. Strains used were DL5743 (Δ*ruvAB*, *lacZ*::246, DSB^+, I-$SceI_{cs}$ across 26 kb),DL5744 (Δ*ruvAB*, $lacZ^+$, DSB^-, I-$SceI_{cs}$ across 26 kb), DL5745 (Δ*ruvAB*, *lacZ*::246, DSB^+, I-$SceI_{cs}$ across 14 kb), DL5746 (Δ*ruvAB*, $lacZ^+$, DSB^-, I-$SceI_{cs}$ across 14 kb), DL5747 (Δ*ruvAB*, *lacZ*::246, DSB^+, I-$SceI_{cs}$ across 16 kb), DL5748 (Δ*ruvAB*, $lacZ^+$, DSB^-, I-$SceI_{cs}$ across 16 kb). Error bars represent the standard error of the mean where n = 3.

https://doi.org/10.1371/journal.pone.0308786.g004

Table 1. Conventional gel electrophoresis conditions that were tested.

	Parameters			
**Conditions**	Agarose concentration	Room temperature or 4˚C	DNA isolated from culture equivalent to OD_{600nm}	Agarose type
	0.8%	Room temperature	6.00	Conventional
	0.5%	Room temperature	6.00	Conventional
		4˚C		
	0.4%	4˚C	6.00	Conventional
	1%	4˚C	6.00	Low Melting Point
	0.4%	4˚C	40.0	Conventional

Running time = 22–24 hours, voltage = 40 volts

https://doi.org/10.1371/journal.pone.0308786.t001

Importantly, these gels did not reveal a DSB-specific band that was able to migrate into the gel. These observations suggest that, like in pulsed-field gel electrophoresis, branched DSBR intermediates formed in large fragments did not migrate into 1% conventional agarose gels but remain trapped within the wells. Also, the strain that was not subjected to DSB, had the DSB region probed fragment in its well. It must also be noted that, as well as the DNA of the probed region that is visible in the blots, there will be a lot of DNA from the rest of the chromosome that will accumulate in these wells indicating that a large percentage of non-specific DNA was trapped in the wells irrespective of DSB.

In order to determine whether some conditions of conventional agarose gel electrophoresis might enable the branched intermediates from a 26 kb fragment to enter the gel, various factors including temperature, agarose concentration and type, and the number of cells from which the DNA was obtained, were investigated (Table 1). DSBR intermediates were confined to the wells under every conditions investigated.

Among the different conditions, 0.4% agarose concentration and 4˚C temperature were chosen since the lower the agarose concentration, the higher the probability of other large DNA fragments coming out of the plug, and gel electrophoresis for a prolonged period of time will generate heat that can melt the gel and therefore low temperature will help in keeping the gel stable.

3.3 Retrieval of the DNA fragments of interest from agarose gel by β-agarase digestion

The observation that DNA recombination intermediates of the desired molecular size does not enter agarose gels under any of the electrophoresis conditions tested led to the second approach. In this approach, a region of synthetic DNA devoid of specific 4-base restriction enzyme recognition sites would enable separation of the DNA of interest retained in the wells of a gel from small digested fragments that can enter the gel. Nevertheless, the recovery of the DNA of interest using the I-SceI digestion approach could be investigated.

The *ruvAB* mutant derivative of the palindrome-containing, SbcCD-inducible strain (DL5743) and an isogenic palindrome free control strain (DL5744) were induced with arabinose for 1 hour. The cells were then treated with psoralen and UV light before being embedded into agarose plugs and lysed. DNA was digested with I-SceI in plugs and run on a 0.4% agarose gel at 4˚C for 22 hours at 40 V. DNA-containing plugs from the well were excised and digested with β-agarase. The extracted DNA was run once more in a 1% agarose gel, transferred to a nylon membrane and probed for the DSB region to visualize the recovery of recombination intermediates (Fig 5).

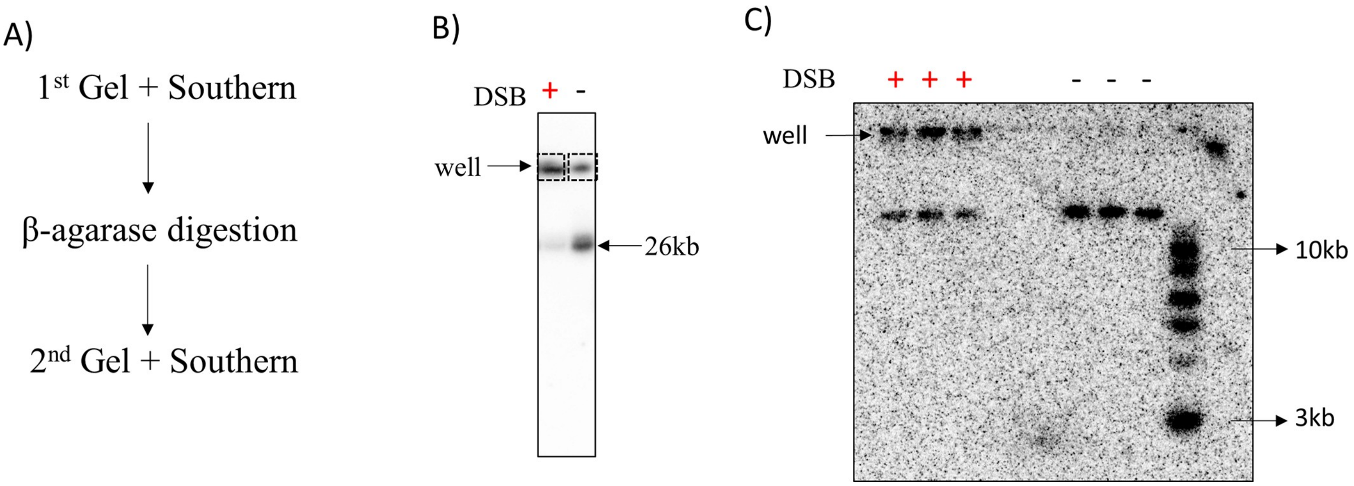

Fig 5. Gel electrophoresis analysis of DNA isolated from the wells of an electrophoresis gel upon β-agarase treatment. A) The steps of the entire protocol are outlined. B) Southern blot of the first electrophoresis gel of the 26 kb DSB region resulting from I-SceI digestion. C) Southern blot of the second electrophoresis gel of the β-agarase treated DNA that was recovered from the well of the first gel. The solution resulting from β-agarase treatment of one agarose plug from the first gel was loaded into three lanes in the second gel. Both DSB$^+$ and DSB$^-$ strains were used. Strains used were DL5743 (Δ*ruvAB*, *lacZ*::246, DSB$^+$), and DL5744 (Δ*ruvAB*, *lacZ*$^+$, DSB$^-$). The marker used was 1 kb DNA ladder from NEB.

https://doi.org/10.1371/journal.pone.0308786.g005

From Fig 5 by measuring the proportion of well DNA content out of total lane in both 1st and 2nd gel electrophoresis (analysis not shown here), it was observed that 90% of the DNA initially trapped in the well of the DSB$^-$ strain migrates as a linear fragment in the second gel. On the other hand, 65% of the DNA initially trapped in the wells of the DSB$^+$ strain is retained in the well in the second gel. These observations are consistent with the formation of recombination intermediates in the DSB$^+$ strain that cannot escape the wells but also trapping of 26 kb linear fragments in the well of the first gel that are freed upon a second round of electrophoresis. This trapping of linear fragments is detected under both DSB$^+$ and DSB$^-$ conditions.

3.4 Optimization of β-agarase treatment

It was investigated whether β-agarase treatment caused damage to extracted DNA fragments. The standard β-agarase protocol involves incubation in a slightly acidic buffer (pH 6.5), posing a risk of nicking DNA [10]. Although crosslinking is expected to hold the molecules together, thereby masking this effect to some extent, it was considered important to minimize DNA nicking of large complex DNA structures to preserve their integrity. Therefore the amount of nicking in the presence of the standard pH 6.5 buffer was investigated and compared to incubation in TAE buffer (pH 8.3).

DSBs were induced in a culture of the *ruvAB* mutant strain DL5743. The culture was split in two, half of the sample was crosslinked with psoralen and UV light, and the second half was not crosslinked. Cells with and without psoralen and UV treatment were embedded in agarose plugs and the DNA was digested with I-SceI in I-SceI reaction buffer (pH 7.9). Following digestion, both sets of plugs were run in an electrophoresis gel. As it has been previously shown that the branched molecules are trapped in the wells, the agarose surrounding the wells was excised from the gel and washed with either only TAE buffer (pH 8.3) or firstly with TAE and then with β-agarase buffer (pH 6.5). The agarose slices were then digested with β-agarase, boiled at 100˚C for a brief period, immediately cooled on ice, and then run on an electrophoresis gel. The probed membrane of the electrophoresis gel is shown in Fig 6.

It can be seen that when the agarose plug containing non-crosslinked DNA was only washed with TAE buffer prior to β-agarase digestion, the resulting DNA solution formed

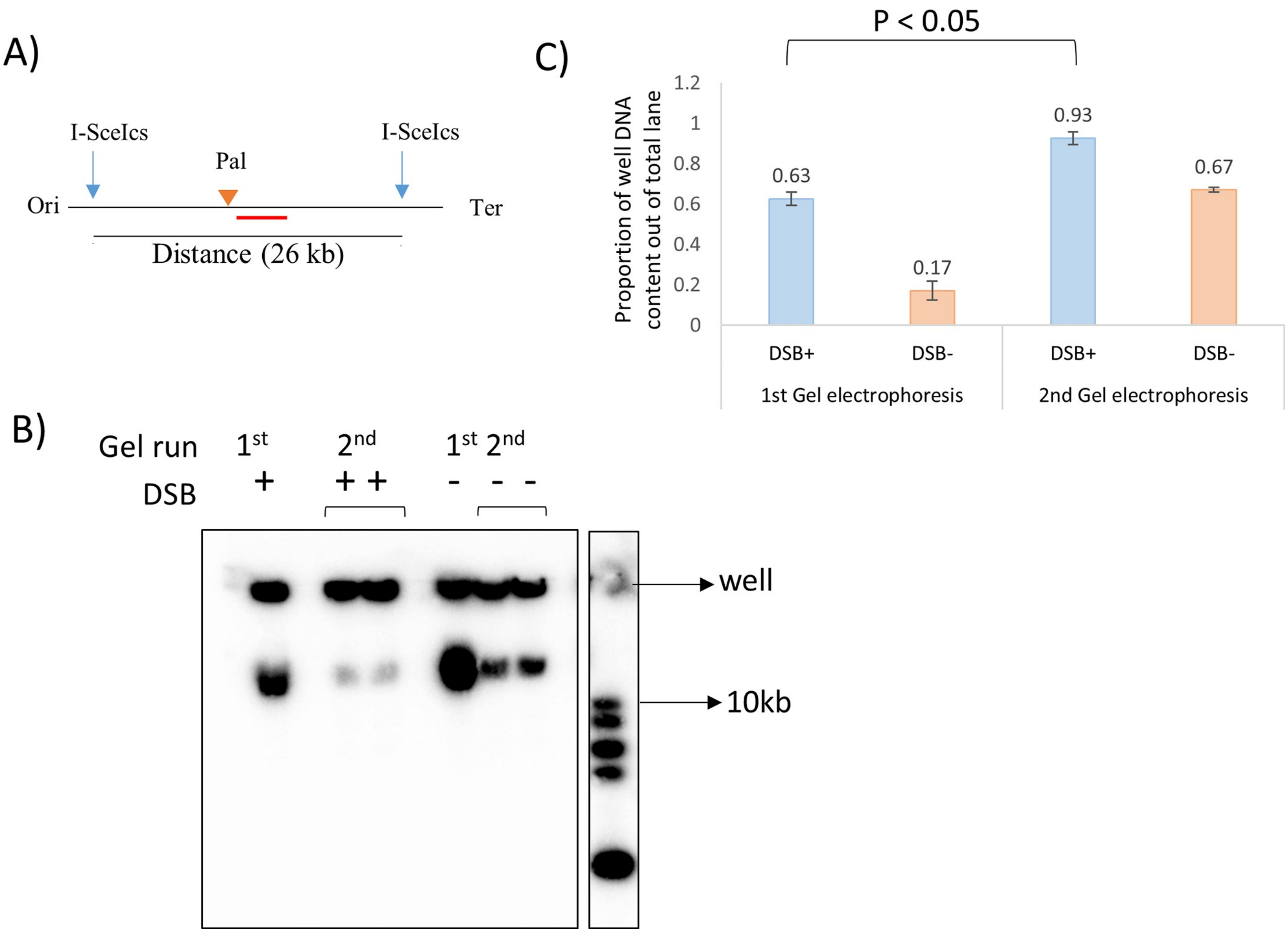

Fig 6. Effects of β-agarase treatment on DNA degradation. Detection of β-agarase treated DNA fragment that was trapped in the agarose plugs after the 1st gel electrophoresis analysis. The first lane represents non-crosslinked DNA fragment containing agarose plug that was washed with only TAE buffer and afterwards boiled without β-agarase treatment, and then run again in this gel. Lanes 2–4 represent non-crosslinked DNA that was washed with only TAE buffer and then treated with β-agarase. The samples in lanes 5–7 are non-crosslinked DNA that was washed with both TAE and β-agarase buffer and then treated with β-agarase. The samples in lanes 8–10 and 11–13 are crosslinked DNA that was equilibrated with only TAE buffer, and TAE followed by β-agarase buffer, respectively, before a β-agarase digestion reaction. The sets of three lanes, containing the DNAs treated similarly, are technical replicates of the same β-agarase treated samples.

https://doi.org/10.1371/journal.pone.0308786.g006

distinct bands on the gel. However, when the agarose plug was washed with both TAE and β-agarase buffer, a smear developed in the gel, suggesting that the DNA had been nicked and that the β-agarase buffer was the cause of this degradation. It is likely that the DNA was depurinated as a result of incubation in the acidic β-agarase buffer. In the instance of crosslinked DNA, the DNA from the agarose plugs was detected in both the wells and the gel following β-agarase digestion. It made no visible difference whether the DNA was equilibrated in TAE buffer or β-agarase buffer. The proportion of the well DNA content was 74% when both TAE and β-agarase buffer was used compared to 76% when only TAE buffer was used. This was interpreted as the presence of crosslinks holding the nicked DNA together. Also, the non-crosslinked DNA formed two linear bands in the gel which represented the ssDNA and dsDNA molecules generated from boiling the sample. In case of crosslinked samples, the ssDNA band was not observed as expected suggesting that the crosslinks were not reversed by boiling.

3.5 Concentrating and purifying DNA recombination intermediates

At this stage, a sample volume of 300–400 μl, derived from pooling the extractions from several agarose plugs, was obtained and it was considered desirable to concentrate it. For instance, a 1–2 μl sample containing 10–50 ng of DNA would be desirable for TEM. However, one needs to be careful to not concentrate unwanted impurities (e.g. undigested agarose and other small particulate matters that would also be visible under the TEM).

A Microcon centrifugal filter was initially used to concentrate the DNA sample. Despite the fact that the sample volume could be lowered to half or one-third of its initial measurement, a white aggregate was found in the recovery tube at the end of the procedure. This aggregate did not form when DNA, that had not been extracted from an agarose gel, was concentrated using the Microcon filter system. Therefore, this aggregate was formed by residual particles from the electrophoresis and needed to be first removed from the sample. Consequently, the sample was first purified by filtration using Sephadex G-25 DNA grade resin in a Microspin column before using the Microcon filter system. This double purification and concentration procedure eliminated the white aggregate, suggesting that this was a useful way of cleaning up the sample following gel extraction using β-agarase.

3.6 Second gel electrophoresis for further purification of branched DNA molecules

Observations made from Fig 4 directed to the next experiment. Using the I-SceI digestion strategy and probes specific for the DSB region (Fig 7A–7C), it was observed that when the well content from a strain not subjected to DSB was run a second time in a gel electrophoresis, around 27% of this well DNA migrated into the gel. In contrast, the same experiment performed on strains attempting to repair DSBs would only let less than 10% of their well DNA content migrate in the second electrophoresis gel.

The data clearly indicate that β-agarase treated DNA recovered from agarose gel wells, shows different migration pattern when again run on a gel, based on the presence or absence of induced DSB in the system. In every gel electrophoresis the difference between DSB+ and DSB- strain's well content was significantly different. In addition,the proportion of well DNA content from DSB+ strains significantly increased after 2nd gel electrophoresis. Only a small proportion of DNA came out into the gel indicating that other non-specific DNAs that were accumulating in the wells of the DSB^{+} strains may come out when run on a gel for the second time.

3.7 An alternative method to isolate DSBR intermediates: Utilization of a synthetic region within the *E. coli* chromosome to isolate recombination intermediates

Branched DSBR intermediates were found to be trapped inside agarose plugs positioned in the wells of gels when I-SceI endonuclease digestion was employed to isolate the DSB region from the remainder of the chromosome. However, they were contaminated with numerous other non-specific DNA molecules that were similarly trapped in the plugs, as a result of their size and shape (Fig 4).

An alternative approach to isolate the DSB region was therefore explored. In this approach (Fig 3B), the rest of the chromosome was digested into small fragments while the DSB region was kept intact. A similar approach has recently been described in *Bacillus subtilis* [11] where genomic DNA was digested into small fragments, using a cocktail of restriction enzymes that do not cut within the fragment of interest containing specific replication intermediates, leaving

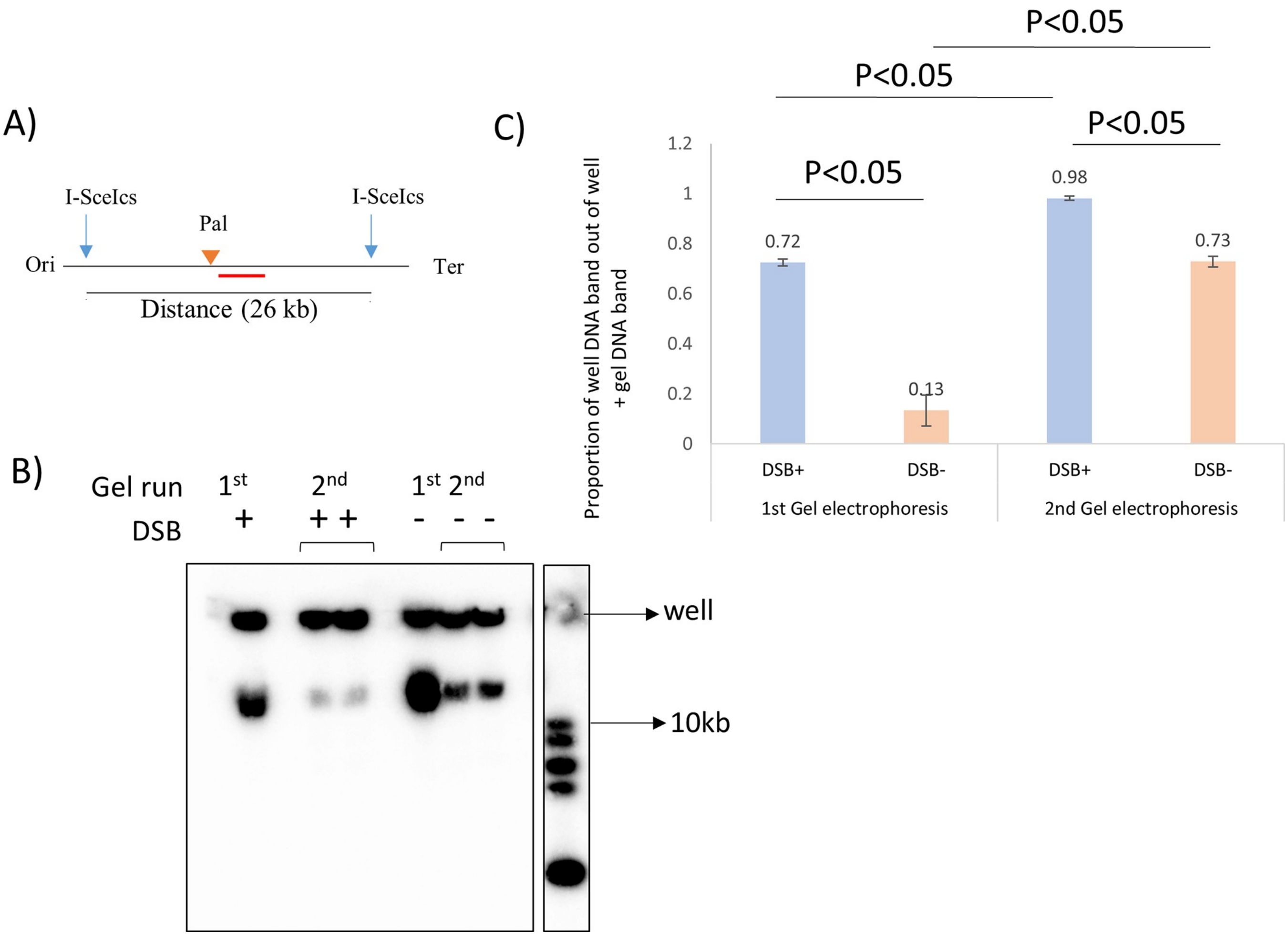

Fig 7. Second gel electrophoresis for further purification of branched DNA molecules. A) I-SceI digestion map of the *E. coli* chromosomal region surrounding the DSB site. The restriction sites and the distance between them are marked with blue vertical arrows and numbers (in kb), respectively. The DSB site is marked by an orange triangle. The radiolabeled probe that was used to detect the fragment is marked by a red line. Ori and Ter mean origin and terminus, respectively. B) Southern blot of a gel electrophoresis where two types of DNA samples are represented including DNA sample undergoing 1st gel electrophoresis and DNA sample recovered from the 1st gel well and undergoing 2nd gel electrophoresis. C) Quantification of the proportion of well DNA content out of the sum of well and gel DNA band after 1st and 2nd gel electrophoreses. The values are shown on the top of each bar. Strains used were DL5743 (Δ*ruvAB*, *lacZ*::246, DSB+), and DL5744 (Δ*ruvAB*, *lacZ*, DSB-). NEB 1 kb DNA ladder was used. Error bars represent the standard error of the mean where n = 3.

https://doi.org/10.1371/journal.pone.0308786.g007

it intact at a larger size than bulk genomic DNA. For this study's purpose a larger region of DNA devoid of restriction targets than was available naturally in the genome was required. A region of synthetic DNA of approximately 51 kb surrounding the DSB site was therefore designed *in silico*. The modified chromosomal region comprised 40 kb of DNA devoid of cutting sites of a set of specific 4-base cutter restriction enzymes (Supplementary Information S1 File: Construction and integration of a 40 kb synthetic region of DNA). The construct carried a kanamycin resistance gene as well as two 5 kb regions with homology to the chromosome for integration by recombination. Once integrated, the rest of the chromosome could be fragmented while 40 kb surrounding the DSB site in the middle would remain intact. Following integration of the synthetic DNA into the chromosome and insertion of the 246 bp palindromic DSB target for SbcCD, it was tested whether digesting the chromosomal DNA with 4-base cutter REs would lead to a better separation of the DSB region from the rest of the

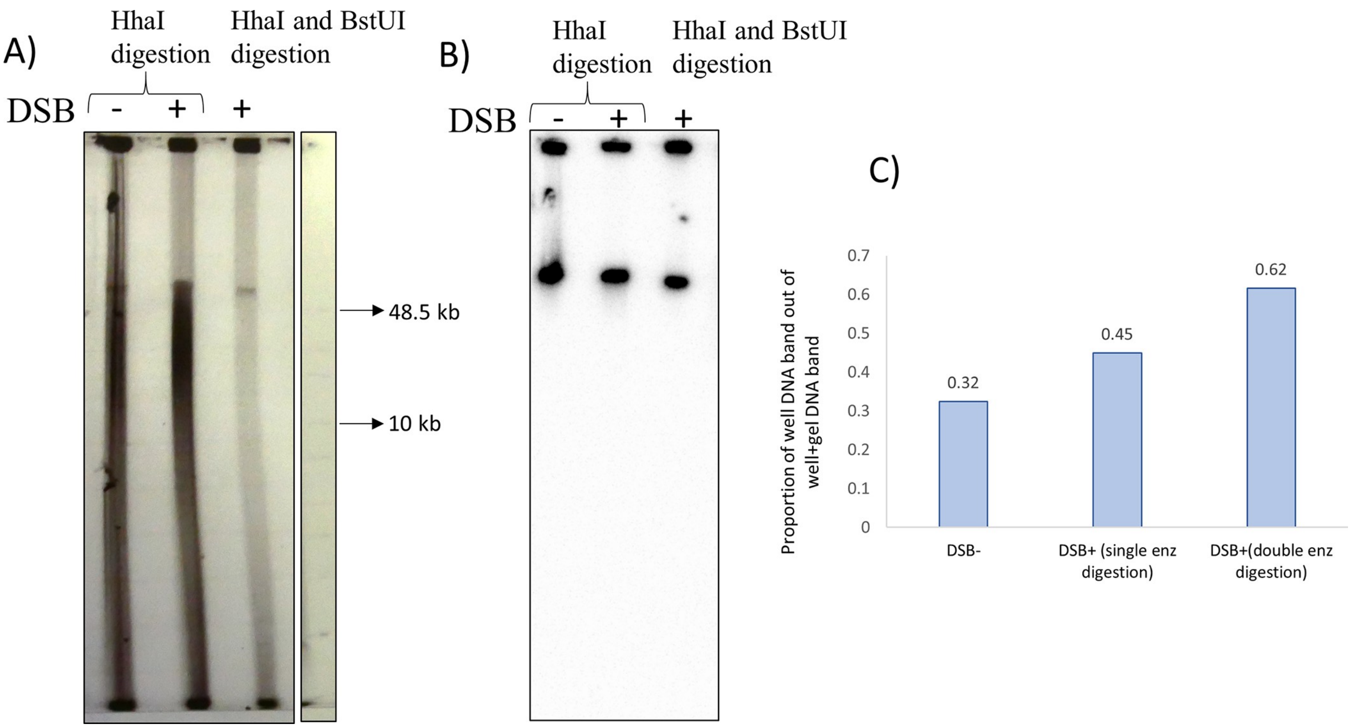

Fig 8. Gel electrophoresis analysis of the synthetic DNA. A) Ethidium bromide staining of a 0.5% agarose gel containing genomic DNA digested with either only HhaI or a combination of HhaI and BstUI. B) Detection of the synthetic DSB region following Southern hybridisation of the gel in A. Both DSB$^+$ and DSB$^-$ cultures of DL7672 were used. A radiolabelled probe was used to detect the synthetic region. The probe did not detect the marker. C) Quantification of the well DNA content out of the total lane is shown.

https://doi.org/10.1371/journal.pone.0308786.g008

chromosome than the I-SceI digestion. Genomic DNA of DL7672, a SbcCD inducible *ruvAB* mutant derivative of the strain containing the synthetic region and the palindrome (with either DSB induced or not induced), was digested overnight with 4-base cutter REs (either HhaI only or HhaI in combination with BstUI), separated by gel electrophoresis and visualized following Southern blotting and hybridisation (Fig 8).

It can be seen (Fig 8A) that the digestion of the DNA with HhaI alone produced a smear that covered nearly the entire length of the gel. Instead, a band that was high in the gel and likely represented the linear DNA fragment containing the DSB site was created when the DNA was digested by a combination of HhaI and BstUI. The identity of this band as the 40 kb linear fragment designed in the synthetic DNA was confirmed by Southern blotting followed by hybridisation with synthetic *lacZ* probe against the DSB region. Due to insufficient replicates, no statistical analyses was performed. Using R package, it was calculated that digestion with each individual restriction enzyme should not produce fragments larger than 2 kb in size. Therefore, it is clear that the double digestion using HhaI and BstUI was more effective in digesting the chromosome than HhaI alone and would reduce the size of the fragments possibly generated from partial digestion by any single enzyme. Ethidium bromide staining revealed the presence of DNA in the wells of the gel irrespective of DSB induction (Fig 8A), and Southern hybridisation showed that while a larger fraction of the synthetic DNA region was present in the wells of the DSB$^+$ strain, it was also present in the wells of the strain that had not undergone DSB (Fig 8B and 8C). These observations demonstrate that DNA accumulates in the wells of the agarose gel for other reasons than branching caused by the induced DSB. This mirrors what was observed when the I-SceI nuclease was used to digest the genomic

DNA (Figs 4 and 6). Following I-SceI digestion, a second round of gel electrophoresis helped to purify the branched intermediates from contaminating DNA (Fig 7). If it would also purify the branched fragments here- was the next question.

3.8 Further purification of the DNA sample

The quality of the DNA after it was concentrated and purified following gel electrophoresis from multiple plugs containing the synthetic region that had undergone DSBR was needed to be assessed. DSBs were induced for 60 minutes using a *ruvAB* mutant containing an arabinose-inducible SbcCD, the synthetic region and a 246 bp palindrome centrally located in the synthetic region (DL7672). The cells were treated with 40 μg/ml of psoralen and UV light to crosslink the DNA. The well DNA from several plugs was concentrated and purified following a gel electrophoresis. The DNA was then run on a 2nd agarose gel, this time not from a plug, but from a liquid solution. The DNA that was still in the second gel wells after running the electrophoresis was extracted using a pipette, and any agarose that might have been present in the wells was digested with β-agarase before concentrating the DNA and purifying it using a G-25 column and a Microcon filter. The quality of that DNA sample was checked by running it in another electrophoresis gel (Fig 9). Ethidium bromide staining revealed that most of the DNA within the sample was still retained in the well, indicating the presence of mainly branched and complex DNA structures. The 40 kb linear DNA band was not detected in the

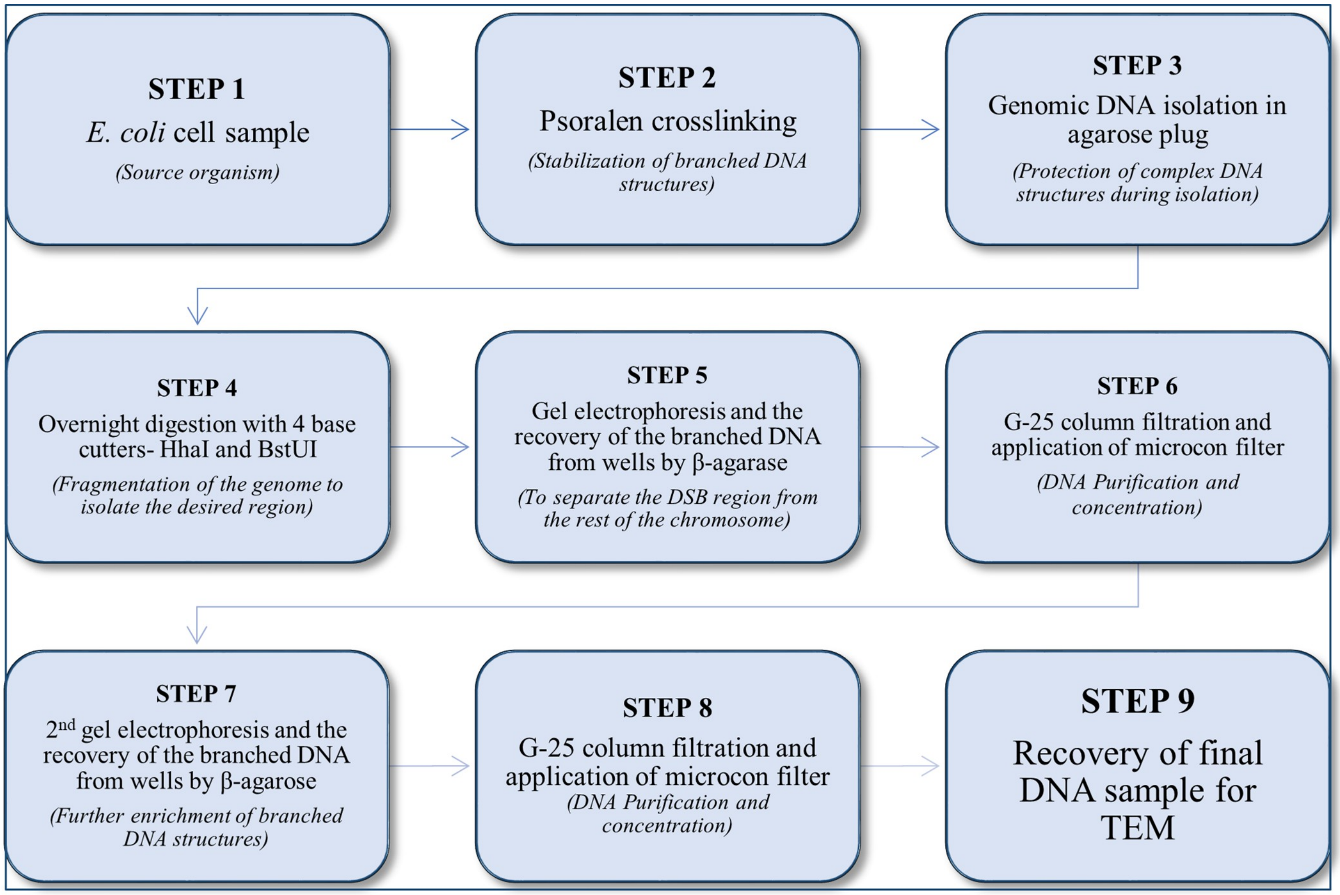

Fig 9. Quality of final DNA sample as depicted by agarose gel electrophoresis and staining following two rounds of gel electrophoresis. Ethidium bromide staining of the gel electrophoresis of β-agarase treated DNA that had undergone DSB and recovered from the wells of a 2nd gel.

https://doi.org/10.1371/journal.pone.0308786.g009

sample, rather only much smaller linear fragments (around 1 kb) could be seen at the bottom of the gel. These observations further indicate that the DNA content still in the wells of a second electrophoresis gel would be a more appropriate sample for EM as it would contain much less non-specific DNA and all the molecules irrespective of being DSB-specific or not, will be visible under the microscope.

These results confirmed the importance of running the DNA on a gel twice in order to maximize the purification of the DNA sample: once to remove the bulk of the linear molecules from the agarose plug and once again to remove the remaining non-specific DNA molecules that were caught inside the agarose plug during the first round of gel electrophoresis.

Raw images are availaible in Supplementary Information S1 Raw images.

4. Discussion

The present understanding of DSBR pathways is largely based on a map of protein activities on simple substrates employed in *in vitro* biochemical studies. However, the large and complex DNA structures envisaged to be formed *in vivo* are mostly absent from one's understanding. Several different models have been proposed in the literature regarding how the HR proteins work *in vivo* but these have not been confirmed at the level of the DNA molecules extracted from live cells. The intricacy of the HR intermediates, the size of the genome, and the capacity to gather significant numbers of intermediates generated at a known genomic region pose the biggest challenges to this knowledge. Even in the context of the comparatively small *E. coli* genome, this is difficult.

This study has described how a protocol was optimized that would allow one to separate and purify DNA recombination intermediates from the rest of the chromosomal DNA to be further analyzed by visualization. The sample was enriched for recombination intermediates that were generated in response to a precisely-located DSB. In order to prevent impurities from obstructing the visualization of the targeted molecules, the molecules required to be thoroughly isolated from the rest of the chromosome and the final sample needed to be as clean as possible. In addition, particular attention was centered on isolating large 20 to 40 kb DNA pieces encompassing the DSBR region to increase the likelihood of finding any potential structures inside this area.

This optimization study revealed information that will be useful to isolate, purify and analyze branched DNA molecules for any analysis including TEM.It was found found that these, branched DNA repair intermediates remain in the wells of agarose gels under all circumstances that weretested. This made it preferable to isolate the molecules of interest by removal of DNA from the rest of the genome using a strategy of digesting the rest of the chromosome in small fragments that would enter a gel (even if they were branched because of structures such as replication forks). This was accomplished by synthesizing *in vitro* a 40kb region surrounding the DSB site and designing it to lack specific 4-base palindromic targets for restriction enzymes that could be used to fragment the rest of the genome.

The extraction from gels by β-agarase digestion, gel filtration and concentration were optimized. The quality of the samples was checked by ethidium bromide staining and autoradiography following Southern blot hybridization. This analysis showed that the sample resulting from a well of a first round of gel electrophoresis still contained non-specific DNA that migrated into the gel as linear fragments when run on a second gel. This may happen because the agarose plug itself hinders the migration of DNA out of the plug. This hypothesis also agrees with the observation that was made with gel electrophoresis of β-agarase treated DNA. In that case, the agarose in the plug was already digested by the enzyme and the resulting DNA solution when run on a gel electrophoresis showed clearly distinguishable migration pattern between DSB^+ and DSB^- strain. It was also observed that the standard β-agarase buffer (pH

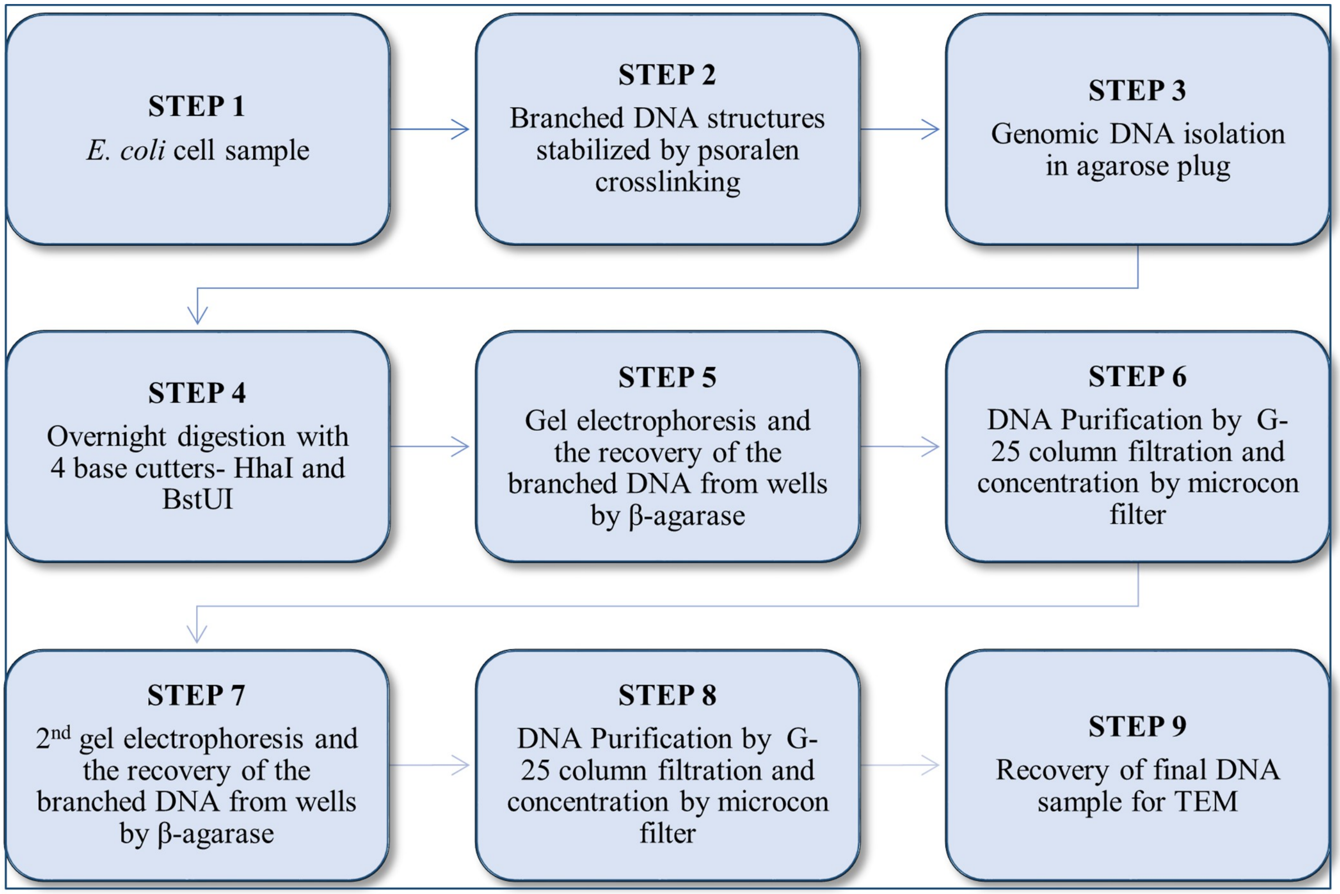

Fig 10. Optimized protocol of sample preparation for studying branched DNA molecules under TEM.

https://doi.org/10.1371/journal.pone.0308786.g010

6.5) caused DNA nicking. However, extraction was possible in TAE buffer (pH 8.3) with no detectable DNA damage. Since β-agarase was equally active in both buffers, TAE buffer was used instead of β-agarase buffer to avoid the nicking.

Summing up these observations, a protocol of the preparation of a DNA sample for visualization by TEM was developed. The outline is given in Fig 10. Resolving the technological difficulties of seeing HR intermediates in *E. coli* is the first step towards comparable investigations in bigger genomes.

5. Conclusion

Branched DNA molecules are the primary molecules of interest for investigating complex molecular mechanisms ranging from DNA synthesis to DNA recombination. The molecular processes can stretch beyond a few kb in length *in vivo* and therefore not only the DNA molecules intricate structures but also their size become crucial factors when one wants to isolate them for investigation. This study has shed light on how to isolate and purify such large, complex DNA structures from genomic DNA.

Supporting information

S1 File. Construction and integration of a 40 kb synthetic region of DNA.
(DOCX)

S1 Table. Bacterial strains, plasmids and oligonucleotides used in this study.
(DOCX)

S1 Raw images.
(PDF)

Acknowledgments

We thank Ewa Okely for technical support, and Elise Darmon and Eva Garcia-Ruiz for their guidance. We also thank Prof. Massimo Lopes of University of Zurich for his valuable suggestions regarding various technical aspects of sample preparation for TEM. TY was granted an EMBO short term fellowship to visit the Lopes Laboratory at the University of Zurich from which she gained many strategical insights into TEM visualization.

Author Contributions

Conceptualization: David R. F. Leach.

Data curation: David R. F. Leach.

Formal analysis: Tahirah Yasmin, David R. F. Leach.

Funding acquisition: David R. F. Leach.

Investigation: Tahirah Yasmin, Benura Azeroglu, Fares Osam Yanez-Cuna, Sally Jones, David R. F. Leach.

Methodology: Tahirah Yasmin, Benura Azeroglu, Fares Osam Yanez-Cuna, Sally Jones, Patrick Yizhi Cai, David R. F. Leach.

Project administration: David R. F. Leach.

Resources: David R. F. Leach.

Supervision: Benura Azeroglu, Patrick Yizhi Cai, David R. F. Leach.

Writing – original draft: Tahirah Yasmin, Sally Jones, David R. F. Leach.

Writing – review & editing: Tahirah Yasmin, Benura Azeroglu, Fares Osam Yanez-Cuna, David R. F. Leach.

References

1. Sinha A.K., Possoz C., Durand A., Desfontaines J.-M., Barre F.-X., Leach D.R.F., et al., Broken replication forks trigger heritable DNA breaks in the terminus of a circular chromosome. PLoS Genetics, 2018. 14(3): p. e1007256. https://doi.org/10.1371/journal.pgen.1007256 PMID: 29522563
2. Vilenchik M.M. and Knudson A.G., Endogenous DNA double-strand breaks: Production, fidelity of repair, and induction of cancer. Proceedings of the National Academy of Sciences, 2003. 100(22): p. 12871–12876.
3. Mawer J.S. and Leach D.R., Branch migration prevents DNA loss during double-strand break repair. PLoS Genet, 2014. 10(8): p. e1004485. https://doi.org/10.1371/journal.pgen.1004485 PMID: 25102287
4. Azeroglu B., Mawer J.S., Cockram C.A., White M.A., Hasan A.M., Filatenkova M., et al., RecG Directs DNA Synthesis during Double-Strand Break Repair. PLoS Genet, 2016. 12(2): p. e1005799. https://doi.org/10.1371/journal.pgen.1005799 PMID: 26872352
5. Vollenweider H.J., Sogo J.M., and Koller T., A routine method for protein-free spreading of double- and single-stranded nucleic acid molecules. Proc Natl Acad Sci U S A, 1975. 72(1): p. 83–7. https://doi.org/10.1073/pnas.72.1.83 PMID: 164030

6. Eykelenboom J.K., Blackwood J.K., Okely E., and Leach D.R., SbcCD causes a double-strand break at a DNA palindrome in the Escherichia coli chromosome. Mol Cell, 2008. 29(5): p. 644–51. https://doi.org/10.1016/j.molcel.2007.12.020 PMID: 18342610

7. Khlebnikov A., Datsenko K.A., Skaug T., Wanner B.L., and Keasling J.D., Homogeneous expression of the P(BAD) promoter in Escherichia coli by constitutive expression of the low-affinity high-capacity AraE transporter. Microbiology (Reading), 2001. 147(Pt 12): p. 3241–7.

8. Yasmin T., Azeroglu B., Cockram C.A., and Leach D.R.F., Distribution of Holliday junctions and repair forks during Escherichia coli DNA double-strand break repair. PLOS Genetics, 2021. 17(8): p. e1009717. https://doi.org/10.1371/journal.pgen.1009717 PMID: 34432790

9. Darmon E., Eykelenboom J.K., Lopez-Vernaza M.A., White M.A., and Leach D.R., Repair on the go: E. coli maintains a high proliferation rate while repairing a chronic DNA double-strand break. PLoS One, 2014. 9(10): p. e110784. https://doi.org/10.1371/journal.pone.0110784 PMID: 25353327

10. An R., Jia Y., Wan B., Zhang Y., Dong P., Li J., et al., Non-Enzymatic Depurination of Nucleic Acids: Factors and Mechanisms. PLOS ONE, 2015. 9(12): p. e115950.

11. Stoy, H., K, S.L., Merrikh H., and Lopes M, Locus-Specific Analysis of Replication Dynamics and Detection of DNA-RNA Hybrids by Immuno Electron Microscopy. Methods Mol Biol, 2022. 2528: p. 67–89. https://doi.org/10.1007/978-1-0716-2477-7_6 PMID: 35704186
